# The Role of Diet in the Cardiovascular Health of Childhood Cancer Survivors—A Systematic Review

**DOI:** 10.3390/nu16091315

**Published:** 2024-04-27

**Authors:** Ruijie Li, Alan R. Barker, Dimitris Vlachopoulos, Dewi Paris, Christina Schindera, Fabiën N. Belle, Raquel Revuelta Iniesta

**Affiliations:** 1 Children’s Health & Exercise Research Centre (CHERC), Faculty of Health and Life Sciences, University of Exeter, Exeter EX1 2LU, UK; rl615@exeter.ac.uk (R.L.); a.r.barker@exeter.ac.uk (A.R.B.); d.vlachopoulos@exeter.ac.uk (D.V.); adp222@exeter.ac.uk (D.P.); 2 Childhood Cancer Research Group, Institute of Social and Preventive Medicine (ISPM), University of Bern, Mittelstrasse 43, 3012 Bern, Switzerland; christina.schindera@unibe.ch (C.S.); fabien.belle@unibe.ch (F.N.B.); 3 Division of Paediatric Oncology/Haematology, University Children’s Hospital Basel, University of Basel, 4056 Basel, Switzerland

**Keywords:** diet, diet recommendations, childhood cancer survivors, cardiovascular diseases, cardiovascular disease risk factors, cardiac dysfunction

## Abstract

Background: Childhood cancer survivors (CCSs) face an increased risk of cardiovascular disease (CVD). This systematic review aims to provide the first synthesis of observational and interventional studies on the relationship between diet and cardiovascular health in CCSs. Methods: A comprehensive search was conducted for studies published between 1990 and July 2023 in PubMed, MEDLINE, CINAHL, Child Development & Adolescent Studies, and Cochrane Library. Eligible studies included observational and interventional studies examining the associations or effects of dietary factors on CVD incidence, cardiac dysfunction, or CVD risk factors in CCSs diagnosed before age 25 years. Results: Ten studies met the inclusion criteria (nine observational and one interventional). Collectively, they comprised 3485 CCSs (male, 1734; female, 1751). The outcomes examined across observational studies included characteristics of obesity, diabetes biomarkers, hypertension indicators, dyslipidaemia biomarkers, and metabolic syndrome. The evidence suggested that greater adherence to healthy diets was associated with lower body mass index, blood pressure, glucose, and triglycerides and higher high-density lipoprotein cholesterol. The 12-week lifestyle intervention study in childhood leukaemia survivors found no impact on obesity indicators. Conclusion: The review results indicate the potentially protective effects of healthy diets. However, the available research remains preliminary and limited, underscoring the need for more rigorous, adequately powered studies.

## 1. Introduction

Worldwide, approximately 400,000 children and adolescents aged 0–19 years develop cancer annually [1]. Notably, over 80% of paediatric cancer patients in high-income countries can be cured due to advancements in therapies and supportive care [1]. Despite this progress, childhood cancer survivors (CCSs) face enduring risks of chronic health complications, with cardiovascular disease (CVD) being the leading non-malignant cause of death, carrying a sevenfold-higher mortality risk compared to peers [2,3]. CVD in CCSs encompasses congestive heart failure, myocardial infarction, coronary artery disease, cardiomyopathy, stroke, pericardial disease, arrhythmias, and valvular and vascular dysfunction, presenting a critical consideration for their physical well-being [4,5]. The incidence of cardiac events in CCSs surpasses that of their siblings by more than two-fold, with an onset typically before the age of 30 years [6,7,8]. Adverse effects on the cardiovascular system stem from the cardiotoxicity of cancer treatments, such as chemotherapy (anthracyclines) and chest-directed radiation therapy, resulting in a prevalence of cardiac dysfunction ranging from 6% to 27% among exposed CCSs [5,9,10,11,12,13]. Risk factors for CVD, including abdominal obesity, hypertension, hyperglycaemia, dyslipidaemia, insulin resistance, and metabolic syndrome, are heightened in CCSs [14,15].

In the general population, the influence of a healthy diet (e.g., rich in fruits and vegetables, legumes, high-fibre and low-glycaemic-index carbohydrates, oily fish, and a moderate-to-low amount of meat/poultry) on CVD risk is well-established, where poor adherence to a healthy diet is linked to unfavourable outcomes in CVD [16,17,18]. Among CCSs, adherence to a healthy diet is of particular importance due to their elevated risk of CVD and premature death compared to the general population. However, adherence to dietary recommendations is suboptimal in this population, marked by the consumption of processed foods high in saturated fat and salt and a low intake of fruits, vegetables, dietary fibre, potassium, and whole grains [19,20,21,22,23,24]. Studies indicate that greater adherence to a Mediterranean diet correlates with reduced CVD risk factors in childhood leukaemia survivors [25], while a more inflammatory, poorer-quality diet is associated with increased risk factors for CVD, including insulin resistance [26].

Although some studies explore the role of diet in CCSs cardiovascular health, limitations such as small sample sizes, insufficient dietary details, and differences in CVD outcome-reporting methods hinder a comprehensive understanding of this relationship. Previous studies used varied dietary assessment methods and reported different diet factors and cardiovascular outcomes. This systematic review is the first to consolidate and evaluate the existing evidence on the relationship between diet and cardiovascular health in CCSs. It aims to comprehensively examine the associations between diet and CVD incidence; cardiac dysfunction; and CVD risk factors, including obesity, hypertension, diabetes, dyslipidaemia, and metabolic syndrome, in CCSs, encompassing both observational and interventional studies.

## 2. Methodology

A protocol was designed and registered on PROSPERO (CRD42023415079). The systematic review process and reporting followed the guidelines of the Cochrane Collaboration [27] and the Preferred Reporting Items for Systematic Reviews and Meta-Analyses (PRISMA) Statement (File S1. PRISMA checklist) [28].

### 2.1. Eligibility Criteria

The inclusion criteria for our study encompass paediatric and adult cancer survivors who were diagnosed with cancer before the age of 25 and have finished treatment, as per the International Classification of Childhood Cancer—3rd edition (ICCC-3) classification. This age cutoff is chosen to focus on participants whose cancer diagnosis occurred during their formative years. In terms of exposure or intervention, we specifically consider dietary factors. This choice is based on the critical role that diet plays in the overall health of CCSs, especially in the context of CVD risk. However, we acknowledge that interventions may include counselling or other lifestyle aspects beyond diet. Therefore, our focus is on any intervention that incorporates dietary components, even as part of a wider holistic lifestyle intervention. We decided to capture the broad spectrum of dietary influences on cardiovascular outcomes in CCSs rather than enforcing a standard control group. The inclusion criteria for outcomes are either CVD conditions, measures of cardiac dysfunction, or CVD risk factors. In terms of study types, interventional studies were included, including randomised controlled trials (RCTs) and non-randomised experimental studies, recognising the need to evaluate the effectiveness of interventions in this population. Additionally, observational studies, including cohort studies, cross-sectional studies, and case-control studies, were incorporated to provide a comprehensive analysis of the relationship between diet and cardiovascular health in CCSs. Case studies, book chapters, guidelines, commentaries, reviews, abstracts, and dissertations were excluded.

### 2.2. Study Outcomes

Our study outcomes encompass a range of cardiovascular events and indicators of cardiac dysfunction, as well as CVD risk factors:

- CVD events, including heart failure, coronary artery disease, myocardial infarction, arrhythmia, cardiomyopathy, stroke, angina pectoris, valvular abnormalities, vascular dysfunction, pericardial disease, and cardiac ischaemia.
- Cardiac dysfunction:
  - Indicators measured by conventional echocardiography:
    - Left ventricular systolic function: left ventricular ejection fraction and shortening fraction.
    - Left ventricular diastolic function: early diastolic left ventricular filling velocity, late diastolic left ventricular filling velocity, early to late left ventricular filling velocity, mitral annular early diastolic velocity, peak mitral flow velocity, peak tricuspid regurgitation velocity, and left atrial maximum volume index.
  - Indicators measured by speckle tracking echocardiography:
    - Left ventricular systolic function: global longitudinal strain, global circumferential strain, and global radial strain.
- Cardiac dysfunction:
  - Characteristics of obesity: body mass index (BMI), waist circumference, waist–hip ratio, percent body fat, visceral adiposity, and subcutaneous adiposity with abdominal computed tomography.
  - Diabetes biomarkers: glucose, insulin, and insulin resistance.
  - Hypertension indicators: blood pressure, pre-hypertension, and hypertension.
  - Dyslipidaemia biomarkers: high-density lipoprotein cholesterol (HDL-C), low-density lipoprotein cholesterol (LDL-C), triglycerides, and dyslipidaemia.
  - Metabolic syndrome: clustering of obesity, hypertension, diabetes, and dyslipidaemia.

### 2.3. Search Strategy and Study Selection

A comprehensive search was conducted for studies published between 1990 and July 2023 in PubMed, MEDLINE (via EBSCOhost), CINAHL (via EBSCOhost), Child Development & Adolescent Studies (via EBSCOhost), and Cochrane Library with no language restrictions. The reference list of all relevant articles and narrative reviews were also examined. The initial search strategy, available at https://www.crd.york.ac.uk/PROSPEROFILES/415079_STRATEGY_20240426.pdf (accessed on 26 April 2024), identified relevant keywords and subject heading searches (MeSH), including “childhood cancer survivors” (children, teenagers/adolescents, and young adult cancer survivors), “diet” (nutrition intake, diet quality), and “cardiovascular health” (cardiac events, cardiac dysfunction, and CVD risk factors). All searches were reiterated prior to the final analysis in July 2023 to ensure the inclusion of all eligible studies.

One researcher (RJ) scanned titles of studies from the electronic search, removing duplicates. Two independent review authors (RJ and DP) then screened the titles and abstracts to exclude ineligible studies. We retrieved the full text of all remaining studies. In instances of disagreement, a third independent reviewer (RRI) made the final decision. The titles and abstracts of the remaining selected studies were scrutinised based on eligibility and exclusion criteria and categorised into “include” and “exclude”. The full text of each study in the “inclusion” category was evaluated by two independent reviewers (RJ and RRI) to ensure alignment with the eligibility criteria. Rayyan software (https://www.rayyan.ai/, accessed on 29 October 2023) was used to screen abstracts and titles [29].

### 2.4. Data Extraction

A data-extraction form was created and initially piloted on 5% of the included articles. Subsequently, the data-extraction form was modified and refined for the full data-extraction process.

The following information were extracted from each eligible study:

- Publication information and study characteristics: title, authors, date of publication, country of publication, study design, study setting, and sample size.
- Population characteristics: sex, race/ethnicity, diagnosis (cancer type), age at childhood cancer diagnosis, age at enrolment, time since diagnosis and/or time since the end of cancer treatment, and cancer treatment history (surgery, chemotherapy, or radiation exposure).
- Study design and methodology: details of diet exposures/interventions, methods of data collection (e.g., questionnaire), outcomes (primary outcomes and secondary outcomes).
- Results: data that can demonstrate the association between diet and cardiovascular health indicators, such as Pearson’s correlation coefficient (r), linear regression analysis (β), and logistic regression (odds ratio). Alternatively, data that can show differences in cardiovascular health under different dietary conditions, such as mean values.

### 2.5. Quality Assessment and Synthesis Methods

The included studies underwent a risk-of-bias assessment using the Joanna Briggs Institute (JBI) risk-of-bias assessment tool [30]. Given the diverse nature of our systematic review, which encompassed both observational studies (including cross-sectional and observational retrospective cohort studies; Supplementary File S2) and interventional studies (comprising both non-randomised experimental studies and randomised controlled trials; Supplementary File S3), the JBI tool provided a comprehensive framework for evaluating methodological quality. The risk-of-bias assessment considered key domains relevant to observational and interventional research, encompassing study design, participant selection, validity and reliability of techniques, clarity of inclusion/exclusion criteria, outcome measures, statistical analyses, and control for confounding factors. Criteria such as the adequacy of randomization, blinding procedures, and the handling of withdrawals and dropouts were also considered for the interventional studies. Each study was independently reviewed by two researchers (RJ and DP) to assess the risk of bias, with particular attention paid to potential sources of systematic error. Quality ratings were assigned as “low risk”, “moderate risk”, or “high risk” to each study, where “low risk” denoted adherence to all aspects of the JBI criteria, “moderate risk” was assigned when there were some concerns without high-risk area, and “high risk” was designated when any high-risk areas were identified. In instances of discrepancy, a third independent reviewer (RRI) resolved any disagreements.

The quality of the evidence for each outcome was assessed by one reviewer (RJ), using the method recommended by the Grading of Recommendations, Assessment, Development, and Evaluation (GRADE) working group [31]. Evidence was categorised into one of four levels of certainty: “high”, “moderate”, “low”, or “very low”. Factors such as a high risk of bias [32], inconsistency in results (unexplained heterogeneity) [33], indirectness of findings (lack of generalizability and/or external validity)[34], imprecision (small sample sizes and/or wide confidence intervals) [35], or identified publication bias [36] led to a downgrade in the certainty of the evidence.

Given the expected heterogeneity across studies in terms of dietary assessment methods, nutrition factors examined, and cardiovascular outcomes reported, a systematic review synthesis approach without a meta-analysis was most appropriate. As noted by Campbell et al. [37], for topics with substantial heterogeneity among studies, a systematic review allows for a rigorous integration of the data while accounting for variabilities across study designs and methodologies. For outcomes with sufficient data, we provide a narrative synthesis summarising the evidence separately for each outcome. We also highlight between-study differences in the dietary exposures and cardiovascular health indicators measured. By systematically reviewing the literature without combining results statistically, we aim to evaluate the breadth of evidence linking diet and cardiovascular outcomes in CCSs, while acknowledging the diversity of factors and measures considered across studies on this emerging topic.

## 3. Results

### 3.1. Study Selection and Characteristics

**Figure 1:**
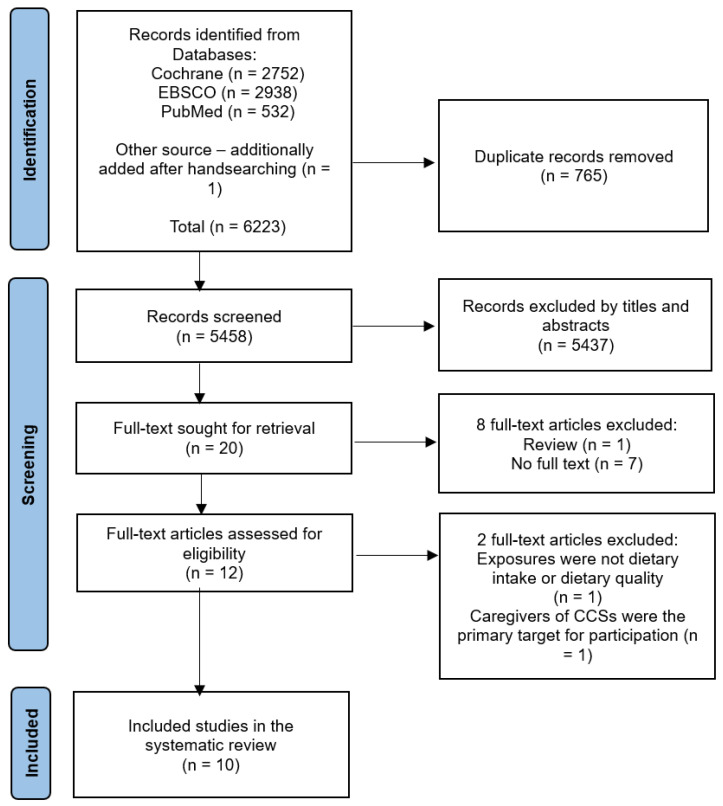
Flow diagram of the studies searched and included in this systematic review.

The flowchart for the selection and inclusion of articles is shown in Figure 1 (PRISMA). Ten studies met the eligibility criteria (Table 1). Of these, seven were cross-sectional studies [22,25,26,38,39,40,41], one was a prospective cohort study [14], one was a retrospective cohort study [42], and one was a non-randomised experimental study [43].

The collective data comprise 3485 CCSs (male, 1734; female, 1751), including both children and young adults, who were initially diagnosed with cancer before reaching the age of 25 years old. Participants in three of the studies were children [38,42,43], three studies involved adults [14,25,41], and four studies included both adults and children [22,26,39,40]. The included studies had a median sample size of 186, ranging from 15 to 1639 participants.

The distribution of study locations was as follows: five studies were conducted in the USA [14,22,25,39,43], three studies in Canada [26,38,40], one study in Switzerland [41], and one study in Turkey [42]. All studies were written in English. In the examination of the relationship between diet and CVD risk factors across nine observational studies, five studies examined food/nutrition intake [38,39,40,41,42], three studies explored diet quality through the use of diet scores [14,25,26], and one study investigated food/nutrition intake—caloric intake and diet quality—Healthy Eating Index (HEI) score [22]. One out of ten studies conducted a non-randomised experimental study [43], involving a 12-week remote lifestyle pilot intervention called Healthy Eating and Active Living (HEAL). The aim of the HEAL study was to prevent excess weight gain among paediatric patients with acute lymphoblastic leukaemia (ALL) undergoing treatment or within two years of treatment completion.

### 3.2. Risk of Bias

**Figure 2:**
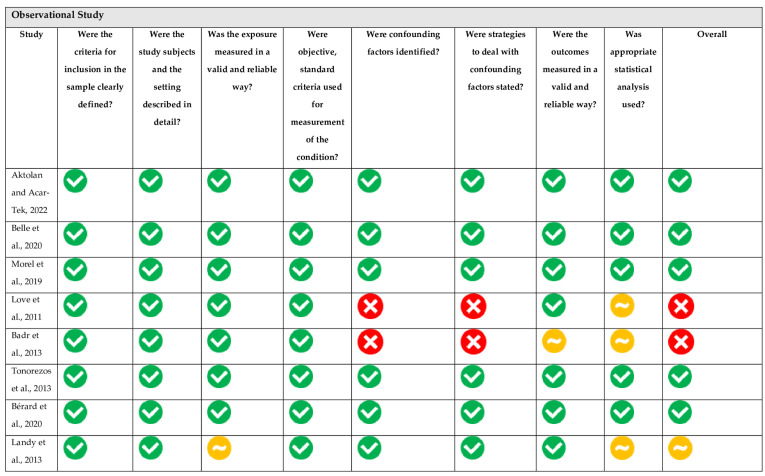

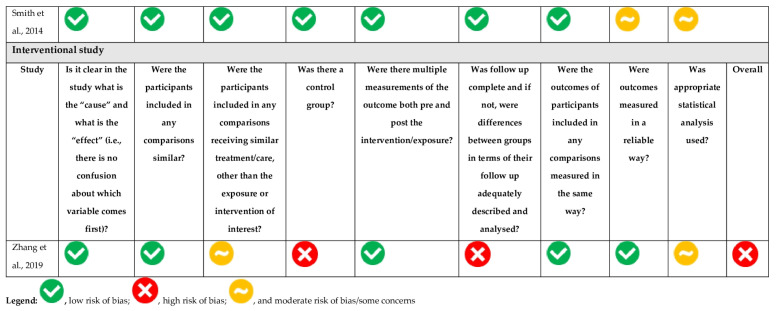
Risk of bias of eligible observational and interventional studies [14,22,25,26,38,39,40,41,42,43].

The risk of bias, using the JBI tool of each study, is summarised in Figure 2. In nine observational studies, two studies were assessed as “high” risk of bias, two “moderate” risk of bias, and five “low” risk of bias. The only interventional study was assessed as “high” risk of bias. The “high” ratings resulted from a lack of consideration for confounding factors. Meanwhile, the “moderate” ratings were mainly attributed to issues with statistical analysis in the “Some Concerns” section. Applying the GRADE methodology, the certainty levels of the outcomes were distributed as follows: three were categorised as “very low”, twelve as “low”, and two as “moderate” (File S4). Predominantly, the evidence was downgraded due to concerns regarding bias, imprecision, and inconsistency within the results.

### 3.3. Outcomes

Nine studies in this review examined the association between nutrition intake/dietary quality and CVD risk factors, including characteristics of obesity (*n* = 8) [14,22,25,26,38,39,41,42], diabetes biomarkers (*n* = 4) [14,22,25,26], hypertension indicators (*n* = 4) [14,22,25,26], dyslipidaemia biomarkers (*n* = 5) [14,22,25,26,40], and cardiometabolic complications (*n* = 4) [14,25,26,41]. No studies were identified reporting cardiac events or cardiac dysfunction as outcomes. Six studies included only ALL survivors [25,26,38,40,42,43], while the populations included in four studies did not restrict the type of childhood cancer [14,22,39,41]. In this systematic review, adhering to guidelines such as the Mediterranean diet; World Cancer Research Fund/American Institute for Cancer Research (WCRF/AICR) guidelines, achieving higher healthy diet scores such as HEI; Mediterranean Diet Quality Index for Children and Adolescents (KIDMED); and Healthy Diet Indicator (HDI), and the consumption of foods beneficial for health are classified as a healthy diet. Conversely, an unhealthy diet is characterised by the excessive energy intake, elevated sodium intake, fast food intake, a more inflammatory diet, consumption of ultra-processed foods, or consumption of foods adverse for health. For a summary, see Table 2.

#### 3.3.1. Associations between Diet and Characteristics of Obesity

Eight out of ten of the included studies investigated the relationship between diet and obesity indicators (Table 2 and Table 3) using BMI (*n* = 7) [22,25,26,38,39,41,42], waist circumference (*n* = 3) [14,25,26], percent body fat (*n* = 1) [22], visceral and subcutaneous adiposity with abdominal computed tomography (*n* = 1) [25], or a combination of BMI ≥ 30 kg/m^2^ in adults and ≥97th percentile in children, waist circumference ≥ 102 cm in men, ≥88 cm in women, and ≥95th percentile in children [26].

Four studies investigated the association between diet and BMI [25,39,41,42]. A significant negative association was reported between adherence to a Mediterranean diet and BMI [25]. CCSs with excessive energy intake tended to be more overweight or obese during remission [42]. However, no association was found between carbohydrate and protein intake and being overweight or obese. Sodium intake estimated from spot urine was positively correlated with BMI [41]. Concerning fibre intake, a marginally negative association with BMI was found [39].

Three studies identified group differences in diet intake when comparing CCS classified as overweight/obese compared to BMI in the normal range [22,38,41]. Further exploration revealed that CCSs classified as obese or overweight had elevated sodium intake compared to those classified as having a normal weight/underweight based on morning-fasting spot urine samples [41]. In another study, ALL survivors were classified as underweight, normal weight, overweight, obese [38]. The overweight group reported the lowest intake of total kilocalories (kcal), fat, and carbohydrates than the normal weight group. However, upon excluding under-reporters, no notable differences in energy intake emerged between the normal weight group and overweight group. Moreover, no association between daily energy intake and BMI, but CCSs who were obese had a lower HEI score compared to their overweight counterparts [22].

Bérard et al. [26] assessed the association between adherence to seven nutritional scores (Mediterranean Diet Adherence Screener (MEDAS), KIDMED, HDI, HEI, Energy-Adjusted Dietary Inflammatory Index (E-DIITM), ferric-reducing ability of plasma (FRAP), and NOVA classification (% UPF)) and BMI, high waist circumference, and obesity [26]. While they observed tendencies suggesting associations between various dietary scores and indicators of adiposity, including BMI and waist circumference, statistical significance was never attained.

Two out of three studies reported that better adherence to healthy diet guidelines was inversely associated with waist circumference [14,25]. Greater adherence to a Mediterranean diet pattern was associated with lower waist circumference [25]. Similarly, a higher prevalence of elevated waist circumference was observed among non-adherent CCSs to the WCRF/AICR [14].

Two studies investigated the relationship between adopting a high-quality diet and body composition, body fat percentage [22], and visceral and subcutaneous fat distribution [25]. The findings revealed no discernible association between daily caloric intake and percent body fat. However, an inverse relationship was observed between total HEI scores and percent body fat [22]. Another study revealed that a greater adherence to a Mediterranean diet was associated with lower visceral and subcutaneous adiposity [25].

#### 3.3.2. Associations between Diet and Diabetes Biomarkers

Table 2 and Table 4 show that 4 out of 10 studies reported diverse evidence concerning the association between diet and diabetes indicators, with a focus on homeostasis model assessment-insulin resistance (HOMA-IR, *n* = 3) [22,25,26], glucose (*n* = 3) [14,22,25], fasting insulin (*n* = 1) [22], and insulin resistance (*n* = 1) [26]. For the latter, insulin resistance was defined as blood fasting glucose ≥ 6.1 mmol/L (109.8 mg/dL), glycated haemoglobin between ≥6% and <6.5%, or a HOMA-IR cutoff of ≥2.86 in adults and ≥95th percentile in children [26].

Two out of three studies found a negative association between a healthy diet with HOMA-IR. Tonorezos et al. [25] investigated the association between adherence to a Mediterranean diet, as assessed by the Mediterranean Diet Score, and HOMA-IR ≥ 2.86. Despite there being no overall association between the Mediterranean Diet Score and HOMA-IR ≥ 2.86, higher dairy intake was inversely associated to elevated HOMA-IR (b = −1.06; *p* ≤ 0.03). However, individual components of the Mediterranean diet, such as meat, alcohol, and fruits/vegetables, showed no significant associations with anthropometric (BMI, waist circumference, and visceral and subcutaneous adiposity) or metabolic outcomes when tested separately. Bérard et al. explored the association between adherence to seven nutritional scores (MEDAS, KIDMED, HDI-2018, HEI-2015, E-DIITM, FRAP, and % UPF) and high HOMA-IR [26]. It was found that high HOMA-IR was associated with a more inflammatory diet, as measured by the E-DII score. However, there were no significant associations between total daily Kcal intake relative to Institute of Medicine (IOM) recommendations or adherence to the HEI with HOMA-IR [22].

Three studies investigate the associations between adherence to healthy diet guidelines with glucose [14,22,25]. CCSs who did not adhere to the guidelines set by the WCRF/AICR were found to have a higher prevalence of elevated fasting glucose [14]. Among CCSs with elevated fasting glucose, 81% of men and 83% of women did not adhere to the WCRF/AICR guidelines. No association was found between blood glucose and total daily Kcal intake or HEI score [22]. Similarly, no association was observed between adherence to a Mediterranean diet and glucose levels ≥ 100 mg/dL [25].

Only one study investigated the relationship between total daily Kcal or HEI score and fasting insulin, but no association was found [22]. Moreover, Bérard et al. [26] investigated insulin resistance, revealing a positive association with a more pro-inflammatory diet as measured by the E-DII score, but these associations did not reach significance.

#### 3.3.3. Associations between Diet and Hypertension Indicators

Of the 10 studies included in the review, 4 reported the association between diet and blood pressure outcomes [14,22,25,26] and 1 reported pre-hypertension/hypertension [26] (see Table 2 and Table 5).

Two out of four studies investigated the association between adherence to healthy diet and blood pressure. Better adherence to a Mediterranean diet, as evidenced by higher Mediterranean Diet Scores, was associated with a lower average systolic and diastolic blood pressure [25]. By contrast, no association between total daily caloric intake relative to IOM recommendations or HEI scores and systolic and diastolic blood pressure was observed [22]. One study reported the prevalence of high blood pressure and adherence to WCRF/AICR guidelines [14]. Among CCSs with high blood pressure, regardless of sex, over 78% of CCSs do not adhere to WCRF/AICR guidelines.

Notable findings emerged in an investigation examining the associations between seven nutritional scores (MEDAS, KIDMED, HDI-2018, HEI-2015, E-DIITM, FRAP, and % UPF) and high blood pressure, as well as pre-hypertension/hypertension, with definitions, respectively, as follows: blood pressure ≥ 130/85 and <140/90 mmHg in adults and ≥90th and <95th percentile for age and height in children and ≥140/90 mmHg or taking medication in adults and ≥95th percentile for age and height or taking medication in children [26]. Better adherence to the KIDMED was negatively associated with the risk of high blood pressure. Conversely, a more inflammatory diet, as indicated by the E-DII score, showed a positive association with high blood pressure and hypertension. Additionally, adherence to the KIDMED and HDI-2018 scores demonstrated that pre-hypertension/hypertension was less likely.

#### 3.3.4. Associations between Diet and Dyslipidaemia Biomarkers

Five out of ten studies investigated the associations between diet and dyslipidaemia biomarkers (Table 2 and Table 6). Of these, five studies used HDL-C as outcome [14,22,25,26,40], two studies used LDL-C [22,26], three studies used triglycerides [14,25,26], and one study used dyslipidaemia [26].

Two out of five studies found that a healthy diet was negatively associated with low HDL-C [25,40]. It is noteworthy that nutrient-specific findings included a significant inverse relationship between low HDL-C and increased intake of proteins, zinc, copper, selenium, riboflavin, and niacin [40]. Meat and fruits also demonstrated protective associations against low HDL-C. Conversely, fast-food intake was associated with an elevated risk. Additionally, adherence to a Mediterranean diet was protective, with a lower risk of low HDL-C corresponding to a higher Mediterranean Diet Score [25]. Bérard et al. [26] expanded the investigation, revealing positive associations between low HDL-C and a more inflammatory diet (E-DII score) and increased consumption of ultra-processed foods (% UPF). A trend towards protection against low HDL was observed with higher MEDAS scores, although it did not reach statistical significance. Furthermore, approximately 81% of CCSs with low HDL did not adhere to WCRF/AICR guidelines [14]. Notably, no significant associations were found between total daily caloric intake or total HEI-2005 score and HDL-C [22].

Two out of three studies found better adherence to healthy diet guidelines having lower high-triglyceride risks. An increased consumption of ultra-processed foods, as indicated by % UPF, demonstrated a positive correlation with elevated triglycerides [26]. In one study, 21% of females and 34% of males had high triglyceride levels [14]. Notably, among CCSs with high triglycerides, 77% of females and 82% of males did not adhere to the WCRF/AICR guidelines. However, no significant association was found between adherence to a Mediterranean diet and triglyceride levels [25].

Two studies investigated the association between diet and LDL-C. These two studies found no significant associations in either the seven nutritional scores (MEDAS, KIDMED, HDI-2018, HEI-2015, E-DIITM, FRAP, and % UPF) [26] or in the total daily caloric intake and total HEI-2005 score [22].

One study explored dyslipidaemia as a composite outcome, defined as triglycerides ≥ 1.7 mmol/L (150.6 mg/dL) in adults or ≥ 1.47 mmol/L (130.2 mg/dL) in children, LDL-C ≥ 3.4 mmol/L (131.5 mg/dL) in adults or ≥ 3.36mmol/L (129.9 mg/dL) in children, or HDL-C < 1.03 in men (39.8 mg/dL), < 1.3 mmol/L (50.3 mg/dL) in women, or < 1.03mmol/L (39.8 mg/dL) in children [26]. Although a higher consumption of ultra-processed foods was positively associated with dyslipidaemia, statistical significance was not reached.

#### 3.3.5. Associations between Diet and Presence of Multiple CVD Risk Factors

Four out of the ten studies reported that diet was associated with the presence of multiple CVD risk factors (Table 2 and Table 7) [14,25,26,41]. Two studies found that better adherence to healthy dietary guidelines (Mediterranean diet and WCRF/AICR) [14,25] is inversely associated with the metabolic syndrome, which was defined as three or more of the following: (1) abdominal obesity (waist circumference of >102 cm in males or >88 cm in females); (2) triglycerides ≥ 150 mg/dL; (3) high-density lipoprotein (HDL) cholesterol < 40 mg/dL in males or <50 mg/dL in females; (4) hypertension (systolic pressure ≥ 130 mm Hg or diastolic pressure ≥ 85 mm Hg); and (5) fasting plasma glucose ≥ 100 mg/dL, consistently across the studies.

One study explored the associations between seven nutritional scores (MEDAS, KIDMED, HDI-2018, HEI-2015, E-DIITM, FRAP, and % UPF) and the presence of two or more CVD risk factors [26]. Specifically, it revealed that a more pro-inflammatory dietary pattern, as indicated by the E-DII score, was associated with an increased risk of experiencing two or more CVD risk factors. Moreover, in the study analysing sodium and potassium intake among CCSs with different CVD risk profiles, findings revealed no distinctions in daily intake based on food frequency questionnaires [41]. However, those with CVD risk factors exhibited a slightly higher sodium intake compared to CVD risk-free CCSs and those with CVD, with obesity playing a notable role in influencing these results.

#### 3.3.6. The Effects of Diet Intervention on Cardiovascular Health

Out of a total of 10 studies examined, only 1 study intervention study was identified. This study focused on a 12-week HEAL pilot intervention in CCSs to investigate the influence of diet on cardiovascular health (Table 8) [43]. The results from this study were limited to indicators related to obesity, namely BMI *Z*-score, BMI percentile, and waist circumference. Following a 12-week intervention involving 15 survivors of ALL, 13 participants successfully completed the programme. Over the course of the intervention, there were two families that did not completed the full program due to a decline in the child’s health or lost to follow-up. The analysis revealed no differences in BMI percentile, BMI Z-score, or waist circumference before and after the intervention.

## 4. Discussion

This is the first systematic review evaluating the evidence of the association and impact of diet on cardiovascular health in CCSs. Under the GRADE framework (Table 2), it can be observed that, except for diabetes, for other CVD risk factors, including obesity, hypertension, dyslipidaemia, and metabolic syndrome, at least 75% or more of the articles showed that better adherence to a healthy diet, whether characterised by specific nutrient intake or adherence to an overall healthy dietary pattern, reported a beneficial association. However, the lack of studies directly assessing cardiac dysfunctions and CVD events limits conclusions regarding the associations or effects of diet on clinical cardiovascular outcomes in this high-risk population. Additionally, only one small pilot study implemented a dietary intervention in CCSs, revealing no changes in obesity measures over 12 weeks. Further research is warranted exploring dietary influences and interventions targeting CVD prevention in CCSs, specifically examining cardiac dysfunctions and CVD events as outcomes.

### 4.1. Associations between Diet and Cardiovascular Health

Across the nine observational studies, various dietary exposures were examined, including nutrients intake [22,38,39,40,41,42] and diet quality/pattern scores [14,22,25,26]. Outcomes centred around established CVD risk factors encompassing characteristics of obesity, diabetes biomarkers, hypertension indicators, dyslipidaemia biomarkers, and the presence of multiple cardiometabolic complications.

Greater consumption of fibre, proteins, phosphorus, sodium, zinc, copper, selenium, riboflavin, niacin, and fruits correlated with lower BMI and higher HDL-C levels [39,40]. Conversely, higher intake of energy, sodium, fat, meat, and fast food were associated with elevated BMI, low HDL-C, and metabolic syndrome risk [38,40,41,42]. In addition to relationships with specific nutrients, research among CCSs demonstrates associations between overall diet quality and cardiometabolic risk. Multiple common indices were utilised to characterise the dietary patterns of CCSs, including the Mediterranean diet, KIDMED, HDI, HEI, E-DIITM, FRAP, % UPF, and the WCRF/AICR guidelines. Although different dietary recommendations/guidelines were included, these dietary recommendations/guidelines are somewhat similar in that they emphasise the intake of vegetables, fruits, whole grains, healthy sources of protein (low-fat dairy products, skinless low-fat poultry, fish/seafood, and nuts), and unsaturated oils, and limit the intake of sweets and red meat. Greater conformity to these healthy dietary patterns was associated with lower BMI [22,25], lower body fat [22], smaller waist circumference [14,25], reduced visceral/subcutaneous fat [25], lower HOMA-IR [26], lower glucose [14], lower blood pressure [14,25,26], higher HDL-C [14,25,26], lower triglycerides [14,26], and lower prevalence of two or more cardiometabolic complications [14,25,26]. These likely reflect the higher content of cardioprotective foods/nutrients (e.g., fibre and antioxidants) and lesser amounts of adverse components (e.g., saturated fat, sodium) typifying these high-quality patterns [44,45].

Similar evidence exists in the non-cancer population regarding the cardioprotective effects of diet. Among the specific aspects of diet explored, fast food consumption was linked to obesity indicators, unfavourable lipid profile, and other cardiometabolic outcomes, due to its high energy content, low nutritional value, and large portion sizes [46,47]. This relationship aligns with existing evidence that fast food, typically high in calories, total and saturated fats, is associated with poor cardiometabolic health [48]. Meanwhile, higher intakes of fibre, vegetables, fruit, and dairy products were associated with lower likelihood of abdominal obesity, insulin resistance, and metabolic syndrome. The fibre and micronutrient content in plant foods may attenuate cardiovascular injury by reducing lipid oxidation, oxidative stress, inflammation, and hypertension [49]. Based on this evidence, reducing refined carbohydrates, processed and red meats, while increasing fibre-rich plant foods and unsaturated fats intake should be advised to both general population and CCSs.

The collective evidence suggests the role of overall diet quality, rather than absolute nutrient quantities, as a key determinant influencing CVD risk in CCSs. This finding not only guides current clinical practices but also shapes the direction of future research, emphasising the need to explore how dietary patterns impact cardiovascular outcomes over isolated nutrient analysis. Moreover, this systematic review reveals that current research often fails to adequately control for confounding. Two observational studies completely omitted confounding factors [38,39]. Among the multiple potential influencers, including clinical factors like age at diagnosis, type of cancer, and cardiotoxic treatment; lifestyle elements such as physical activity, smoking, and alcohol assumption; and general characteristics like age, sex, education level, and household income, only four studies accounted for four or more of these factors [14,40,41,42]. Adjustments for physical activity, smoking, and alcohol consumption are less frequently made in the included studies.

Additionally, there is a lack of studies examining associations with cardiac dysfunction and cardiovascular events in CCSs. This limits conclusions regarding the influence of diet on cardiac dysfunction and cardiovascular event risk. The exclusive focus on surrogate markers also precludes determining the effect size of dietary impact on hard endpoints. Nonetheless, despite not translating directly into event reductions, the modulation of risk factors is likely meaningful. Targeting factors such as obesity, dyslipidaemia, and hypertension through diet is a crucial prevention strategy, given the premature onset and aggressive course of CVD in CCSs [7]. Dietary associations with risk factors additionally highlight potential areas of intervention. Ultimately, adequately powered investigations with long-term follow-up are required to clarify diet relationships with definitive cardiovascular endpoints, incorporating better control of confounding factors to enhance the validity of the findings.

### 4.2. The Effects of Diet Intervention on Cardiovascular Health

Beyond observational research, only one pilot study implemented and evaluated a 12-week dietary intervention in CCSs as a means of reducing CVD risk [43]. In the lone interventional trial reviewed, the HEAL program incorporated key components of established lifestyle interventions, including positive parenting style and practices, healthy eating, and physical activity. Notably, compliance and retention in lifestyle interventions is a notable challenge in this population. Qualitative research suggests that some survivors prefer to move beyond their cancer experience and perceive participation in ongoing health research as a reminder of their illness [50]. Therefore, adopting less medicalised approaches could enhance both recruitment and sustained engagement. In the group of 15 participants included, 13 were on maintenance therapy, and 2 post-treatment completion (within two years). Study findings revealed no significant changes in obesity indicators, including BMI and waist circumference, over 12 weeks. Conclusions cannot be derived from a single non-randomised pilot trial with a small sample size at high risk of bias. Additionally, the intervention had a combined focus on promoting both healthy eating and physical activity behaviours. Isolating the effects of healthy eating was not feasible. Nonetheless, this pilot research provides a useful basis to inform future dietary and cardiovascular risk reduction studies in survivors of childhood cancers. Implications for practice include consideration of less intensive or clinical trial-oriented study designs, as well as family-based interventions supporting healthy behaviours.

Currently, there is still a lack of targeted experiments on the cardiovascular health effects of nutritional interventions for CCSs. Several studies have demonstrated the positive impact of nutritional interventions on cardiovascular health in the non-cancer population. A systematic review of behavioural interventions for obese adults without a history of childhood cancer with additional risk factors for morbidity reported consistent modest improvements in behaviour, weight loss, and cardiovascular disease risk factors over time, especially for interventions targeting both diet and physical activity [51]. Furthermore, research has shown that dietary modifications, including adherence to the Mediterranean diet, can lead to improved cardiovascular health, as evidenced by the positive effects on endothelial progenitor cells, which are surrogate markers for evaluating cardiovascular health [52]. Nutritional interventions have positive effects on cardiovascular health, but specific evidence regarding the impact on the cardiovascular health of CCSs still requires targeted intervention experiments.

Ultimately, the dearth of interventional research underscores the necessity of well-designed, adequately powered randomised controlled trials investigating the causal effects of diet on CVD risk in CCSs. Future interventions can emphasise personalised guidance and behaviour change strategies, including goal setting, self-monitoring, and frequent interaction with health coaches. Clarifying effective approaches through rigorous evaluation can direct the translation of lifestyle programs to attenuate elevated cardiovascular risk in this population. Findings would additionally inform lifestyle guidelines and policies for CCSs. See future research recommendations in Box 1. The recommendations may encounter significant practical challenges, including the need to control for various confounding factors, secure long-term follow-up, and gather sufficiently large sample sizes. These aspects entail substantial financial and logistical commitments. Nevertheless, addressing these challenges is essential for acquiring robust and clinically relevant data. We emphasise that, despite these difficulties, pursuing this line of research is crucial for a comprehensive understanding of the impact of nutrition on cardiovascular health among childhood cancer survivors. Future studies should consider these factors to enhance the validity and applicability of their findings.

Box 1 **Recommendations for future research.**

1. To perform high-quality national and/or international observational studies in which confounding factors are accounted for, power calculations are performed and where appropriate (longitudinal studies) length of follow-up is reported.
2. Examine associations of specific foods/nutrients, rather than overall diet quality, with cardio-metabolic risk factors to further clarify nutritional needs of survivors.
3. Investigate barriers and facilitators to adopting healthy lifestyles in diverse groups of childhood cancer survivors to inform targeted behavioural interventions.

### 4.3. Strength and Limitations

This systematic review possesses several key strengths. Firstly, it is the first to synthesise global evidence regarding diet and cardiovascular health specifically among CCSs. The focus on survivors is valuable given their elevated risks compared to peers, warranting investigation of modifiable factors like nutrition. Secondly, the broad search strategy facilitated capturing all relevant studies meeting eligibility criteria across multiple databases without exclusions based on date, country, or language. Thirdly, the quantitative synthesis helped identify overarching trends across the heterogeneous data by delineating findings by common outcomes and dietary exposures. The qualitative risk-of-bias assessment highlighted study limitations to aid contextualised interpretation. The reporting adheres to PRISMA systematic review guidelines, enhanced clarity and reproducibility.

However, some limitations to this review warrant consideration. The considerable heterogeneity across studies in exposures, outcomes, and statistical methodologies precluded meta-analysis. Therefore, we could not quantitatively integrate results or assess for publication bias. In addition, relatively few studies met the eligibility criteria, indicating a nascent evidence base requiring expansion. Moreover, the predominance of cross-sectional observational data prohibits determining causality. Additionally, investigations utilised inconsistent dietary assessment methodologies with varying validity and reliability. Furthermore, definitions of exposures like “healthy eating” and “dietary guidelines” differed across studies, complicating integration and comparison. There was also heterogeneity in outcomes examined and their definitions. Moreover, adjustment for potential confounders was inconsistent. Finally, populations were mostly North American and European, with limited diversity, and survivors predominately comprised ALL patients, reducing generalizability to other diagnoses.

## 5. Conclusions

This systematic review synthesises current evidence assessing diet relationships with cardiovascular outcomes among CCSs. The observational data demonstrate that better adherence to healthy dietary recommendations is associated with lower CVD risk factors, but intervention research is severely lacking. Encouraging survivors and their families to adhere to health diet pattern emphasising whole grains, fruits/vegetables, legumes, nuts, and healthy fats early in the survivorship period may alleviate CVD risk-factor rates. However, the potential for nutritional strategies to prevent clinical CVD endpoints in CCSs remains unproven, marking a critical knowledge gap warranting investigation. Large cohort studies tracking detailed dietary intake alongside cardiac dysfunction and cardiac events are needed to directly quantify associations and establish evidence-based nutritional guidelines specifically for CCSs. Given the complex and synergistic interplay between lifestyle factors and cancer treatments in pathways underlying CVD progression, randomised controlled trials are imperative to determine causal effects of dietary improvement initiatives on CVD risk factors. The findings will inform the development of tailored, guidelines-driven nutrition programs within survivorship care plans promoting lifelong cardiovascular health and longevity in CCSs.

## Figures and Tables

**Table 1:** Publication information, study characteristics, population characteristics, study aims, and study design of all eligible studies.

Author and Country	Study Design	Participants	Age at Cancer Diagnosis	Age at Enrolment	Diagnosis	Study Aims	Exposures/Interventions	Outcome—CVD-Relevant Variables	Risk of Bias
(Love et al., 2011) [38] Canada	Cross-sectional study	102 children (male, 47; female, 55)	Median age, 3.3 years (range, 0.4–11.1 years)	Median age, 14.3 years (range, 8.4–18.6 years)	ALL	Examined the relationship between BMI and demographic and lifestyle factors in a cohort of ALL survivors.	Food/nutrient intake Fat intake (g) Protein intake (g) Carbohydrate intake (g) Calorie intake (kcal)	BMI	High
(Badr et al., 2013) [39] USA	Cross-sectional study	170 (male, 88; female, 82)	Mean age, 9.1 years (SD, 5.5) (range, 0.27–20.1 years)	Mean age, 17.7 years (SD 5.6) (range, 3.3–28.9 years)	CNS Leukaemia Lymphoma Sarcoma	Characterize the relationship between weight status (i.e., BMI) and lifestyle behaviours (i.e., diet and physical activity) among CCSs and determine whether differences in weight status and lifestyle behaviours exist depending on group-level characteristics.	Food/nutrient intake Fruit and vegetable intake Fibre intake Energy from fat	BMI	High
(Landy et al., 2013) [22] USA	Cross-sectional Study	91 survivors (Male 42, Female 49) 30 siblings (Male 18, Female 12). Control group: 30 siblings	Survivors 0 to 5 years, 42 (46%); 6 to 12 years, 29 (32%); 13 years or older, 20 (22%)	Survivors 5 to 12 years, 21 (23%); 13 to 18 years, 20 (22%); 19 to 29 years, 34 (38%); 30 years or older, 16 (18%)	Leukaemia Brain cancer Sarcoma Lymphoma Other	Examine whether specific cancer diagnoses or therapies are associated with diet and how diet is related to adiposity and traditional CVD risk factors among survivors.	Diet quality/Diet Score Daily caloric Total HEI score	BMI Waist circumference Percent body fat High HOMA-IR Glucose Insulin Systolic blood pressure Diastolic blood pressure Low HDL-C High LDL-C	Moderate
(Tonorezos et al., 2013) [25] USA	Cross-sectional Study	117 adults (male, 52; female, 65)	Mean age, 6.7 years (SD, 4.3)	Median age 24.3 years (SD, 4.9) (range, 18–37 years)	ALL	Determine the relationship between diet and metabolic abnormalities among adult survivors of childhood ALL.	Diet quality/Diet Score Mediterranean Diet Score	BMI Waist circumference Visceral adiposity Subcutaneous adiposity High HOMA-IR Glucose Low HDL-C Triglycerides Metabolic syndrome	Moderate
(Smith et al., 2014) [14] USA	Prospectively cohort study	1639 adults (female, 832; male, 807)	Mean age, 7.9 years (SD, 5.5)	Median age, 32.7 years (range, 18.9–60.0 years)	Leukaemia Lymphoma Sarcoma Neuroblastoma Wilms tumour CNS Other	Characterise lifestyle habits and associations with metabolic syndrome among CCSs.	Diet quality/Diet Score WCRF/AICR guidelines	Metabolic syndrome	Moderate
(Morel et al., 2019) [40] Canada	Cross-sectional study	Total, 241: 156 adults (57.3% male); 85 children (49.4% male)	Median age, 4.7 years (range, 0.9–18.0 years)	Median age, 21.3 years (range, 8.5–40.9 years)	ALL	This study aimed to examine the associations between food/nutrient intake and the levels of HDL-C in a cohort of children and young adult survivors of ALL.	Food/nutrient intake Energy intake (kcal) Macronutrients: Proteins (g) Carbohydrates (g) Dietary fibre (g) Lipids (g) Omega-6 (g) Omega-3 (g) Ratio w-6/w-3 Micronutrients: Calcium (mg) Iron (mg) Magnesium (mg) Phosphorus (mg) Potassium (mg) Sodium (mg) Zinc (mg) Copper (mg) Manganese (mg) Selenium (mcg) Retinol (mcg) Folic acid (mcg) Niacin (mg) Riboflavin (mg) Thiamine (mg) Vitamin B6 (mg) Vitamin B12 (mcg) Choline (mg) Vitamin C (mg) Vitamin D (mcg) Vitamin K (mcg) Food groups: Meat Fish and seafood Dairy Fat Vegetables Legumes Fruits	Low HDL-C	Low
(Zhang et al., 2019) [43] USA	Interventional study	15 children (male, 11; female, 4) 13 (86.7%) completed No control group	N/R	Mean age, 6.1 years (SD, 2.0) (range, 3.8–9.8 years)	ALL	Preventing excess weight gain among patients with paediatric ALL who were on treatment or within two years of treatment completion.	A 12-week lifestyle intervention delivered remotely through web-based sessions and phone calls with a lifestyle coach—HEAL intervention.	BMI Waist circumference	High
(Belle et al., 2020) [41] Switzerland	Cross-sectional study	802 CCSs with FFQ data (female, 401; male, 401) Sent 212 a spot urine sample collection kit. 111 morning-fasting spot urine samples (52%) were returned.	Median age, 9.7 years (range, 3.9–13.9 years)	Median age, 34.6 years (range, 28.8–41.1 years)	Leukaemia Lymphoma CNS tumour Neuroblastoma Retinoblastoma Renal tumour Hepatic tumour Bone tumour Soft tissue sarcoma Germ cell tumour Other tumours Langerhans cell histiocytosis	Assessed sodium (Na) and potassium (K) intake, estimated from FFQ and morning urine spots, and its associations with cardiovascular risk in CCSs.	Food/nutrient intake Sodium (Na) Potassium (K)	BMI CVD CVD risk factors	Moderate
(Bérard et al., 2020) [26] Canada	Cross-Sectional Study	Total, 241: 156 adults (49.4% male); 85 children (49.4% male)	Median age 4.7 years (range, 0.9–18.0 years)	Median age, 21.3 years (range, 8.5–40.9 years)	ALL	Explores the associations between diet quality indices, cardiometabolic health indicators and inflammatory biomarkers among ALL survivors.	Diet quality/Diet Score MEDAS KIDMED HDI-2018 HEI-2015 E-DIITM FRAP % UPF	BMI Waist circumference Obesity High HOMA-IR Insulin resistance High blood pressure Hypertension Low HDL-C High LDL-C Triglycerides Dyslipidaemia 2 or more cardiometabolic complications	Low
(Aktolan and Acar-Tek, 2022) [42] Turkey	Observational retrospective cohort study	67 children (boys, 35; girls, 32)	Median age 4.5 years (range 1–13 years)	Median age, 9.7 years (range, 5–15 years)	ALL	Determine the prevalence and related factors of obesity/abdominal obesity and evaluate the association between nutrition and overweight/obesity after cancer treatment in paediatric ALL survivors.	Food/nutrient intake Energy Carbohydrate Protein	BMI	Low

Abbreviations: ALL, acute lymphoblastic leukaemia; BMI, body mass index; CCSs, childhood cancer survivors; CNS, central nervous system; CVD, cardiovascular disease; E-DIITM, Energy-Adjusted Dietary Inflammatory Index; FFQ, food frequency questionnaire; FRAP, ferric-reducing ability of plasma; HDI, Healthy Diet Indicator; HDL-C, high-density-lipoprotein cholesterol; HEI, Healthy Eating Index; HEAL, Healthy Eating and Active Living; HOMA-IR, homeostasis model of insulin resistance; KIDMED, Mediterranean Diet Quality Index for Children and Adolescents; LDL-C, low-density-lipoprotein cholesterol; MEDAS, Mediterranean Diet Adherence Screener; SD, standard deviation; % UPF, NOVA classification; WCRF/AICR, World Cancer Research Fund/American Institute for Cancer Research.

**Table 2:** Summary of results between diet and CVD risk factors for all outcomes.

	Outcome	Participants (Studies) ^a^	Certainty of the Evidence (GRADE)	Key Finding
Obesity indicators	BMI	1635 (8)	㊉㊉㊀㊀	Inverse association between healthy diet (Mediterranean diet [25], fibre intake [39]) and BMI in 2 studies. Positively association between unhealthy diet (excessive energy intake [42], sodium intake [41]) and BMI in 2 studies. Dietary differences (sodium [41], total kilocalories and carbohydrates [38], HEI score [22]) existed in the obese/overweight and normal BMI groups in 3 studies. NS in 2 observational studies (carbohydrate and protein intake [42], seven nutritional scores (MEDAS, KIDMED, HDI, HEI, E-DIITM, FRAP, % UPF) [26]). NS in 1 interventional study [43].
	Waist circumference	2012 (4)	㊉㊉㊀㊀	Inverse association between healthy diet (Mediterranean diet) and waist circumference in 1 study [25]. Not adhere to the WCRF/AICR guidelines have a higher prevalence of elevated waist circumference in 1 study [14]. NS in 1 observational study (seven nutritional scores (MEDAS, KIDMED, HDI, HEI, E-DIITM, FRAP, % UPF)) [26]. NS in 1 interventional study [43].
	Percent body fat	121 (1)	㊉㊀㊀㊀	Inverse association in 1 study (HEI score) [22].
	Visceral adiposity and subcutaneous adiposity	117 (1)	㊉㊀㊀㊀	Inverse association in 1 study (Mediterranean diet) [25].
	Obesity ^b^	241 (1)	㊉㊉㊀㊀	NS in 1 observational study (seven nutritional scores (MEDAS, KIDMED, HDI, HEI, E-DIITM, FRAP, % UPF)) [26].
Diabetes indicators	HOMA-IR	479 (3)	㊉㊉㊀㊀	Inverse association between healthy diet (Mediterranean diet) and high HOMA-IR in 1 study [25]. Positively association between unhealthy diet (inflammatory diet—E-DII score) and high HOMA-IR in 1 study [26]. NS in 1 observational study (total daily Kcal intake and HEI score) [22].
	Glucose	1877 (3)	㊉㊉㊀㊀	Not adhere to the WCRF/AICR guidelines have a higher prevalence of elevated fasting glucose [14]. NS in 2 observational studies (total daily Kcal intake or HEI score [22], Mediterranean diet [25]).
	Insulin	121 (1)	㊉㊀㊀㊀	NS in 1 observational study (total daily Kcal or HEI score) [22].
	Insulin resistance ^c^	241 (1)	㊉㊉㊀㊀	NS in 1 observational study (seven nutritional scores (MEDAS, KIDMED, HDI, HEI, E-DIITM, FRAP, % UPF)) [26].
Hypertension indicators	Blood pressure	2118 (4)	㊉㊉㊀㊀	Inverse association between healthy diet (Mediterranean diet [25], KIDMED [26]) and high blood pressure in 2 studies. Positively association between unhealthy diet (inflammatory diet - E-DII score) and high blood pressure in 1 study [26]. Not adhere to the WCRF/AICR guidelines have a higher prevalence of high blood pressure in 1 study [14]. NS in 1 observational study (total daily caloric intake and HEI score) [22].
	Hypertension ^d^	241 (1)	㊉㊉㊀㊀	Inverse association between healthy diet (KIDMED and HDI-2018 scores) and hypertension in 1 study [26].
Dyslipidaemia indicators	HDL-C	2359 (5)	㊉㊉㊀㊀	Inverse association between healthy diet (Mediterranean diet [25], nutrients intake: proteins, zinc, copper, selenium, riboflavin, niacin, meat, fruits [40]) and low HDL-C in 2 studies. Positive associations between unhealthy diet (inflammatory diet (E-DII score) and ultra-processed foods (% UPF) [26], fast food intake [40]) and low HDL-C in 2 studies. Not adhere to the WCRF/AICR guidelines have a higher prevalence of low HDL-C in 1 study [14]; NS in 1 observational study (total daily caloric intake or HEI score) [22].
	LDL-C	362 (2)	㊉㊉㊀㊀	NS in 2 observational studies (total daily caloric intake and HEI score [22], seven nutritional scores (MEDAS, KIDMED, HDI-2018, HEI-2015, E-DIITM, FRAP, % UPF) [26]).
	Triglycerides	1997 (3)	㊉㊉㊀㊀	Positively associations between unhealthy diet (ultra-processed foods (% UPF)) and high triglycerides in 1 study [26]. Not adhere to the WCRF/AICR guidelines have a higher prevalence of high triglycerides in 1 study [14]. NS in 1 observational study (Mediterranean diet) [25].
	Dyslipidaemia ^e^	241 (1)	㊉㊉㊀㊀	NS in 1 observational study (seven nutritional scores (MEDAS, KIDMED, HDI-2018, HEI-2015, E-DIITM, FRAP, % UPF)) [26].
Presence of multiple CVD risk factors	Presence of 2 or More CVD risk factors	1043 (2)	㊉㊉㊉㊀	Positively associations between unhealthy diet (inflammatory diet - E-DII score) and presence of two or more CVD risk factors in 1 study [26]; Slightly higher sodium intake in CCSs with CVD risk factors than CVD risk-free CCSs [41].
	Metabolic syndrome ^f^	1756 (2)	㊉㊉㊉㊀	Inverse association between healthy diet (Mediterranean diet [14] and WCRF/AICR [25]) and metabolic syndrome in 2 studies.

Abbreviations: BMI, body mass index; E-DIITM, Energy-Adjusted Dietary Inflammatory Index; FRAP, ferric-reducing ability of plasma; HDI, Healthy Diet Indicator; HDL-C, high-density-lipoprotein cholesterol; HEI, Healthy Eating Index; HOMA-IR, homeostatic model assessment—insulin resistance; KIDMED, Mediterranean Diet Quality Index for Children and Adolescents; LDL-C, low-density-lipoprotein cholesterol; MEDAS, Mediterranean Diet Adherence Screener; NS, not significant; % UPF, NOVA classification; WCRF/AICR, World Cancer Research Fund/American Institute for Cancer Research. ^a^ Participants (studies): number of participants (number of studies) included. ^b^ Obesity was defined as having at least one of the following: BMI ≥ 30 kg/m^2^ in adults and ≥97th percentile in children, waist circumference ≥ 102 cm in men, ≥88 cm in women and ≥95th percentile in children. ^c^ Insulin resistance was defined as having at least one of the following: blood fasting glucose ≥6.1 mmol/L (109.8 mg/dL), glycated haemoglobin ≥ 6% and <6.5% and homeostasis model assessment-insulin resistance ≥ 2.86 in adults and ≥95th percentile in children. ^d^ Hypertension was defined, respectively, as blood pressure ≥ 130/85 and <140/90 mmHg in adults and ≥90th and <95th percentile for age and height in children and ≥140/90 mmHg or taking medication in adults and ≥95th percentile for age and height or taking medication in children. ^e^ Dyslipidaemia was defined as having at least one of the following: triglycerides ≥ 1.7 mmol/L (150.6 mg/dL) in adults and ≥1.47 mmol/L (130.2 mg/dL) in children, LDL-C ≥ 3.4 mmol/L (131.5 mg/dL) in adults and ≥3.36 mmol/L (129.9 mg/dL) in children, HDL-C < 1.03 in men (39.8 mg/dL), and <1.3 mmol/L (50.3 mg/dL) in women and <1.03 mmol/L (39.8 mg/dL) in children. ^f^ Metabolic syndrome was defined as three or more of the following: (1) abdominal obesity (waist circumference of >102 cm in males or >88 cm in females); (2) triglycerides ≥ 150 mg/dL; (3) high-density lipoprotein (HDL) cholesterol < 40 mg/dL in males or <50 mg/dL in females; (4) hypertension (systolic pressure ≥ 130 mm Hg or diastolic pressure ≥ 85 mm Hg); and (5) fasting plasma glucose ≥ 100 mg/dL, consistently across the studies. Certainty of Evidence classified as either “very low”, “low”, “moderate”, or “high”. The certainty could be downgraded due to a high risk of bias, inconsistency (unexplained heterogeneity), indirectness (lack of generalisability or external validity), imprecision (small sample size or wide confidence intervals), or the presence of publication bias. ㊉㊀㊀㊀ = very low, ㊉㊉㊀㊀ = low, ㊉㊉㊉㊀ = moderate, ㊉㊉㊉㊉ = high.

**Table 3:** Associations between diet and obesity indicators.

Study	Exposures/ Interventions	Outcomes	Data Analysis Method	Confounding (Method)	Results				
					BMI	Waist Circumference	Percent Body Fat	Visceral Adiposity and Subcutaneous Adiposity	Obesity
Love et al., 2011 [38]	Fat intake (g) Protein intake (g) Carbohydrate intake (g) Calorie intake (kcal)	Participants were classified as underweight (BMI < 5th percentile), normal weight (5th to < 85 percentile), overweight (85th to 95th percentile), or obese (≥95th percentile). Calorie and macronutrient intake by weight category (whole cohort; under-reporters excluded).	Multiple regression	N/R	Mean in whole cohort: Fat (g): normal weight 74.7, overweight 60.2, ∆14.5, *p* = 0.02. Protein: normal weight 85.6, overweight 80. Carbohydrate (g): normal weight 281.7, overweight 242.2, ∆39, *p* = 0.05 Calories (kcal): normal weight 2126.7, overweight 1802.7, ∆324, *p* = 0.018 Mean in under reports excluded: Fat (g): normal weight 84.2, overweight 88 Protein: normal weight 90.8, overweight 106.1 Carbohydrate (g): normal weight 314.7, overweight 320.4 Calories (kcal): normal weight 2364.7, overweight 2472	N/A	N/A	N/A	N/A
Badr et al., 2013 [39]	Fruit and vegetable intake (servings/day) Fibre intake (g/day) Energy from fat (%)	Association between food intake and BMI.	Pearson correlations	N/R	Fibre intake: r = −0.15, *p* = 0.10.	N/A	N/A	N/A	N/A
Landy et al., 2013 [22]	Daily caloric intake Total HEI scores	Association between daily caloric intake relative to IOM recommendations or HEI scores and BMI.	F test and linear regression	Age Sex	Daily caloric intake: F(2) = 0.52, *p* = 0.60 Total HEI scores: F(2) = 2.51, *p* = 0.09	N/A	Daily caloric intake: β = −0.05, *p* = 0.59 Total HEI scores: β = −0.19, *p* = 0.04.	N/A	N/A
Tonorezos et al., 2013 [25]	Mediterranean Diet Score	The relationship between adherence to a Mediterranean diet, measured by the Mediterranean Diet Score, and BMI (≥25 kg/m^2^), waist circumference (>88 cm in women; >102 cm in men), visceral and subcutaneous adiposity.	Logistic regression and linear regression	Age Sex	Logistic regression, OR (95% CI): Mediterranean Diet Score 4–5: 0.3 (0.1–0.9) Mediterranean Diet Score 6–8: 0.3 (0.1–1.1) (*p* = 0.04) Linear regression: β = −1.05, *p* = 0.004.	Logistic regression, OR (95% CI): Mediterranean Diet Score 4–5: 0.4 (0.1–1.0) Mediterranean Diet Score 6–8: 0.2 (0.1–0.7) (*p* = 0.003) Linear regression: β = −2.17, *p* = 0.005.	N/A	Linear regression: Visceral adiposity: β = −0.3, *p* = 0.007. Subcutaneous adiposity: β = −0.11, *p* = 0.001.	N/A
Smith et al., 2014 [14]	WCRF/AICR guidelines	Associations between meeting WCRF/AICR guidelines and waist circumference.	Log-binomial regression	Age Race CRT (medical records) Education (questionnaires) Smoking status (questionnaires) Age at diagnosis (medical records)	N/A	Greater than 40% (41.6%) of the women had an elevated waist circumference, 87.0% of whom were not adherent to the WCRF/AICR guidelines. 29.9% of man had an elevated waist circumference, 91.3% of whom were not adherent to the WCRF/AICR guidelines.	N/A	N/A	N/A
Belle et al., 2020 [41]	Na intake estimated from FFQ Na intake estimated from spot urine K intake estimated from FFQ K intake estimated from spot urine	The correlation between BMI and sodium and potassium measurements by food frequency questionnaire (FFQ) and morning-fasting spot urine samples. Mean sodium (Na) and potassium (K) intake (g/day) in childhood cancer survivors by self-repot BMI (Obese BMI > 30 kg/m^2^, Overweight 25 kg/m^2^ < BMI < 30 kg/m^2^, Normal/underweight BMI < 25 kg/m^2^), retrieved from ANCOVA.	Correlation	Sex Age at survey ICCC-3 cancer diagnosis (medical records) Education level (questionnaires) Smoking habits (questionnaires) Physical activity (questionnaires) Diet quality (Modified AHEI) Alcohol consumption (FFQ)	Na intake estimated from spot urine: r = 0.57, *p* < 0.05 Na intake estimated from FFQ: r = 0.16, *p* = N/R K intake estimated from spot urine: r = 0.04, *p* = N/R K intake estimated from FFQ: r = 0.03, *p* = N/R Mean Na intake based on FFQ (*p* = 0.355) Obese: 2.9 (95% CI 2.8–2.9) Overweight: 2.9 (95% CI 2.8–2.9) Normal/underweight: 2.8 (95% CI 2.8–2.9) Mean K intake based on FFQ (*p* = 0.296) Obese: 2.6 (95% CI 2.3–2.8) Overweight: 2.8 (95% CI 2.6–2.9) Normal/underweight: 2.8 (95% CI 2.7–2.9) Mean Na intake based on morning-fasting spot urine (*p* < 0.001) Obese: 4.2 (95% CI 3.8–4.6) Overweight: 3.3 (95% CI 3.0–3.6) Normal/underweight: 2.7 (95% CI 2.6–2.9) Mean K intake based on morning-fasting spot urine (*p* = 0.272) Obese: 2.1 (95% CI 1.5–2.7) Overweight: 1.6 (95% CI 1.1–2.1) Normal/underweight: 1.5 (95% CI 13–1.7)	N/A	N/A	N/A	N/A
Bérard et al., 2020 [26]	MEDAS KIDMED HDI-2018 HEI-2015 E-DIITM FRAP % UPF	Association between the seven nutritional scores and high BMI, high waist circumference and obesity. Obesity was defined as having at least one of the following: BMI ≥ 30 kg/m^2^ in adults and ≥ 97th percentile in children, waist circumference ≥ 102 cm in men, ≥88 cm in women and ≥ 95th percentile in children.	Logistic regression	Sex Survival time (medical records)	No statistically significant association between the seven dietary scores and high BMI (no *p* < 0.05). Tertile 2 vs. 1, OR (95% CI) MEDAS: 1.114 (0.38–3.20), *p* = 0.84 KIDMED: 1.147 (0.15–8.68), *p* = 0.90 HDI-2018: 1.259 (0.49–3.25), *p* = 0.63 HEI-2015: 1.225 (0.46–3.24), *p* = 0.68 E-DIITM: 1.297 (0.50–3.34), *p* = 0.59 FRAP: 1.372 (0.52–3.59), *p* = 0.51 % UPF: 0.360 (0.10–1.24), *p* = 0.11 Tertile 3 vs. 1, OR (95% CI) MEDAS: 0.990 (0.32–3.11), *p* = 0.99 KIDMED: 1.010 (0.12–8.42), *p* = 0.89 HDI-2018: 0.811 (0.30–2.21), *p* = 0.63 HEI-2015: 1.162 (0.44–3.10), *p* = 0.76 E-DIITM: 1.260 (0.47–3.41), *p* = 0.65 FRAP: 0.556 (0.19–1.67), *p* = 0.52 % UPF: 0.929 (0.34–2.58), *p* = 0.89 Tertile 2 and 3 vs. 1, OR (95% CI) MEDAS: 1.004 (0.39–2.57), *p* = 0.99 KIDMED: 1.056 (0.17–6.52), *p* = 0.95 HDI-2018: 0.949 (0.41–2.20), *p* = 0.90 HEI-2015: 1.295 (0.55–3.06), *p* = 0.56 E-DIITM: 1.280 (0.55–3.01), *p* = 0.57 FRAP: 0.946 (0.39–2.33), *p* = 0.90 % UPF: 0.619 (0.25–1.54), *p* = 0.30	No statistically significant association between the seven dietary scores and high waist circumference (no *p* < 0.05). Tertile 2 vs. 1, OR (95% CI) MEDAS: 0.374 (0.14–1.04), *p* = 0.06 KIDMED: 0.429 (0.12–1.50), *p* = 0.18 HDI-2018: 0.983 (0.48–2.02), *p* = 0.96 HEI-2015: 0.859 (0.43–1.74), *p* = 0.67 E-DIITM: 1.089 (0.54–2.18), *p* = 0.81 FRAP: 1.259 (0.62–2.56), *p* = 0.53 % UPF: 0.622 (0.28–1.37), *p* = 0.24 Tertile 3 vs. 1, OR (95% CI) MEDAS: 0.671 (0.25–4.12), *p* = 0.44 KIDMED: 0.638 (0.20–2.00), *p* = 0.44 HDI-2018: 1.161 (0.57–2.35), *p* = 0.68 HEI-2015: 0.770 (0.38–1.57), *p* = 0.47 E-DIITM: 1.574 (0.76–3.26), *p* = 0.22 FRAP: 0.994 (0.46–2.17), *p* = 0.99 % UPF: 0.968 (0.44–2.13), *p* = 0.94 Tertile 2 and 3 vs. 1, OR (95% CI) MEDAS: 0.470 (0.19–1.14), *p* = 0.09 KIDMED: 0.646 (0.23–1.82), *p* = 0.41 HDI-2018: 1.002 (0.54–1.85), *p* = 1.00 HEI-2015: 0.774 (0.41–1.42), *p* =0.41 E-DIITM: 1.283 (0.69–2.37), *p* = 0.43 FRAP: 1.144 (0.60–2.19), *p* = 0.69 % UPF: 0.772 (0.39–1.51), *p* = 0.45	N/A	N/A	No statistically significant association between the seven dietary scores and obesity (no *p* < 0.05). Tertile 2 vs. 1, OR (95% CI) MEDAS: 0.599 (0.23–1.53), *p* = 0.29 KIDMED: 0.429 (0.12–1.49), *p* = 0.19 HDI-2018: 1.039 (0.51–2.10), *p* = 0.92 HEI-2015: 0.884 (0.44–1.77), *p* = 0.73 E-DIITM: 1.137 (0.57–2.27), *p* = 0.72 FRAP: 1.212 (0.60–2.44), *p* = 0.59 % UPF: 0.578 (0.26–1.27), *p* = 0.17 Tertile 3 vs. 1, OR (95% CI) MEDAS: 0.799 (0.30–2.10), *p* = 0.65 KIDMED: 0.638 (0.20–2.00), *p* = 0.44 HDI-2018: 1.093 (0.54–2.20), *p* = 0.80 HEI-2015: 798 (0.40–1.61), *p* = 0.53 E-DIITM: 1.686 (0.82–3.46), *p* = 0.16 FRAP: 0.904 (0.42–1.95), *p* = 0.80 % UPF: 1.002 (0.46–2.17), *p* = 1.00 Tertile 2 and 3 vs. 1, OR (95% CI) MEDAS: 0.571 (0.25–1.31), *p* = 0.19 KIDMED: 0.646 (0.23–1.82), *p* = 0.41 HDI-2018: 1.240 (0.70–2.21), *p* = 0.46 HEI-2015: 0.801 (0.44–1.46), *p* = 0.47 E-DIITM: 1.356 (0.74–2.50), *p* = 0.33 FRAP: 1.075 (0.57–2.04), *p* = 0.83 % UPF: 0.759 (0.39–1.47), *p* = 0.41
Aktolan and Acar-Tek, 2022 [42]	Energy Carbohydrate Protein	The subjects were classified into four categories of BMI for age z score (BAZ): underweight (≤−2 SD to −1 SD), normal weight (–1 SD to 1 SD), overweight (1 SD to 2 SD), and obese (≥2 SD). Logistic regression models constructed to examine energy, carbohydrate, and protein for being overweight or obese in remission.	Logistic regression	Sex Age at diagnosis (medical records) Receipt of CRT (medical records) Treatment risk category (medical records)	OR (95% CI), *p*-value Excessive energy: 3.217 (0.181–8.761), *p* = 0.022 Excessive carbohydrate: 0.615(0.210–1.800) *p* = 0.375 Excessive Protein: 0.402 (0.150-.,077) *p* = 0.7	N/A	N/A	N/A	N/A

Abbreviations: AHEI, Alternative Healthy Eating Index; ANCOVA, Analysis of Covariance; BMI, body mass index; CI, confidence interval; CRT, cranial radiotherapy; E-DIITM, Energy-Adjusted Dietary Inflammatory Index; FFQ, food frequency questionnaire; FRAP, ferric-reducing ability of plasma; HDI, Healthy Diet Indicator; HEI, Healthy Eating Index; ICCC-3, International Classification of Childhood Cancer, 3rd edition; IOM, Institute of Medicine; KIDMED, Mediterranean Diet Quality Index for Children and Adolescents; MEDAS, Mediterranean Diet Adherence Screener; N/A, not applicable; N/R, not reported; OR, odd ratio; % UPF, NOVA classification; WCRF/AICR, World Cancer Research Fund/American Institute for Cancer Research.

**Table 4:** Association between diet and diabetes biomarkers.

Study ID	Exposures/ Interventions	Outcomes	Data Analysis Method	Confounding (Method)	Results			
					HOMA-IR	Glucose	Insulin	Insulin Resistance
Tonorezos et al., 2013 [25]	Mediterranean Diet Score	The relationship between adherence to a Mediterranean diet, measured by the Mediterranean Diet Score, and HOMA-IR ≥ 2.86 and Glucose ≥ 100 mg/dl.	Logistic regression	Age Sex	OR (95% CI): Mediterranean Diet Score 4–5: 1.5 (0.6–3.8) Mediterranean Diet Score 6–8: 0.6 (0.2–1.6) (*p* = 0.36) Higher dairy intake was associated with higher HOMA-IR [insulin resistance, (b = −1.06; *p* = 0.029)].	OR (95% CI): Mediterranean Diet Score 4–5: 3.5 (0.9–14.5) Mediterranean Diet Score 6–8: 0.7 (0.1–1.6) (*p* = 0.96)	N/A	N/A
Landy et al., 2013 [22]	Daily caloric intake, Total HEI score	Associations between total daily caloric intake relative to IOM recommendations or HEI scores and individual CVD risk factors including HOMA-IR, glucose, insulin.	Multivariate linear regression	Age Sex	Daily caloric intake: β = 0.19, *p* = 0.10 Total HEI score: β = 0.00, *p* = 0.97	Daily caloric intake: β = 0.05, *p* = 0.63 Total HEI score: β = 0.08, *p* = 0.48	Daily caloric intake: β = 0.18, *p* = 0.11 Total HEI score: β = 0.00, *p* = 0.97	N/A
Smith et al., 2014 [14]	WCRF/AICR guidelines	Associations between meeting WCRF/AICR guidelines and fasting glucose.	Log-binomial regression	Age Race CRT (medical records) Education (questionnaires) Smoking status (questionnaires) Age at diagnosis (medical records)	N/A	Of the 38.2% of men with elevated fasting glucose, 80.8% were not adherent to the WCRF/AICR guidelines. 24.9% of women with elevated fasting glucose, 83.1% were not adherent to the WCRF/AICR guidelines.	N/A	N/A
Bérard et al., 2020 [26]	MEDAS KIDMED HDI-2018 HEI-2015 E-DIITM FRAP % UPF	Association between adherence to nutritional scores and high HOMA-IR and insulin resistance. Insulin resistance was defined as having at least one of the following: blood fasting glucose ≥ 6.1 mmol/L (109.8 mg/dL), glycated haemoglobin ≥ 6% and <6.5% and homeostasis model assessment-insulin resistance ≥ 2.86 in adults and ≥95th percentile in children.	Logistic regression	Sex Survivor time (medical records)	Tertile 2 vs. 1, OR (95% CI) MEDAS: 1.621 (0.59–4.48), *p* = 0.35 KIDMED: 0.675 (0.14–3.37), *p* = 0.63 HDI-2018: 0.894 (0.35–2.26), *p* = 0.81 HEI-2015: 1.660 (0.69–3.98), *p* = 0.26 E-DIITM: 2.667 (1.11–6.43), *p* = 0.03 FRAP: 0.897 (0.38–2.13), *p* = 0.81 % UPF: 0.341 (0.11–1.03), *p* = 0.06 Tertile 3 vs. 1, OR (95% CI) MEDAS: 0.760 (0.23–2.47), *p* = 0.65 KIDMED: 0.629 (0.12–3.28), *p* = 0.58 HDI–2018: 1.302 (0.55–3.11), *p* = 0.55 HEI–2015: 0.947 (0.37–2.44), *p* = 0.91 E-DIITM: 1.349 (0.50–3.68), *p* = 0.56 FRAP: 0–540 (0.20–1.45), *p* = 0.22 % UPF: 0.763 (0.30–1.97), *p* = 0.58 Tertile 2 and 3 vs. 1, OR (95% CI) MEDAS: 0.741 (0.30–1.84), *p* = 0.50 KIDMED: 0.615 (0.15–2.51), *p* = 0.50 HDI–2018: 1.109 (0.51–2.41), *p* = 0.79 HEI–2015: 1.207 (0.55–2.64), *p* = 0.64 E-DIITM: 2.047 (0.89–4.70), *p* = 0.09 FRAP: 0.733 (0.33–1.64), *p* = 0.45 % UPF: 0.533 (0.23–1.23), *p* = 0.14	N/A	N/A	A more pro–inflammatory diet was positively associated with insulin resistance and hypertension, but results were not statistically significant. (no *p* < 0.05). Tertile 2 vs. 1, OR (95% CI) MEDAS: 1.641 (0.59–4.55), *p* = 0.34 KIDMED: 0.675 (0.14–3.37), *p* = 0.63 HDI-2018: 0.903 (0.36–2.28), *p* = 0.83 HEI-2015: 1.746 (0.73–4.16), *p* = 0.21 E-DIITM: 2.144 (0.93–4.95), *p* = 0.07 FRAP: 0.917 (0.39–2.17), *p* = 0.84 % UPF: 0.508 (0.19–1.39), *p* = 0.19 Tertile 3 vs. 1, OR (95% CI) MEDAS: 1.040 (0.34–3.21), *p* = 0.95 KIDMED: 0.629 (0.12–3.28), *p* = 0.58 HDI-2018: 1.512 (0.64–3.56), *p* = 0.34 HEI-2015: 1.017 (0.40–2.58), *p* = 0.97 E-DIITM: 1.095 (0.42–2.87), *p* = 0.85 FRAP: 0.695 (0.27–1.80), *p* = 0.45 % UPF: 0.800 (0.31–2.06), *p* = 0.64 Tertile 2 and 3 vs. 1, OR (95% CI) MEDAS: 0.845 (0.35–2.07), *p* = 0.71 KIDMED: 0.615 (0.15–2.51), *p* = 0.50 HDI-2018: 1.212 (0.56–2.61), *p* = 0.62 HEI-2015: 1.284 (0.59–2.79), *p* = 0.53 E-DIITM: 1.650 (0.75–3.62)), *p* = 0.21 FRAP: 0.817 (0.37–1.81), *p* = 0.62 % UPF: 0.642 (0.28–1.46), *p* = 0.29

Abbreviations: CI, confidence interval; CRT, cranial radiotherapy; CVD, cardiovascular disease; E-DIITM, Energy-Adjusted Dietary Inflammatory Index; FRAP, ferric-reducing ability of plasma; HDI, Healthy Diet Indicator; HEI, Healthy Eating Index; HOMA-IR, homeostasis model of insulin resistance; subjects were categorised as being insulin resistant when HOMA-IR was equal to or more than 2.86 (above the 75th percentile for HOMA-IR derived from the Third National Health and Nutrition Examination Survey); IOM, Institute of Medicine; KIDMED, Mediterranean Diet Quality Index for Children and Adolescents; MEDAS, Mediterranean Diet Adherence Screener; N/A, not applicable; OR, odd ratio; % UPF, NOVA classification; WCRF/AICR, World Cancer Research Fund/American Institute for Cancer Research.

**Table 5:** Association between diet and hypertension indicators.

Study ID	Exposures/ Interventions	Outcomes	Data Analysis Method	Confounding (Method)	Results	
					Blood Pressure	Hypertension
Landy et al., 2013 [22]	Daily caloric intake Total HEI score	Associations between total daily caloric intake relative to IOM recommendations or HEI scores and systolic and diastolic blood pressure.	Multivariate linear regression	Age Sex	Systolic blood pressure: Daily caloric intake β = 0.18, *p* = 0.09 Total HEI score β = −0.05, *p* = 0.61 Diastolic blood pressure: Daily caloric intake β = −0.09, *p* = 0.41 Total HEI score: β = 0.05, *p* = 0.59	N/A
Tonorezos et al., 2013 [25]	Mediterranean Diet Score	The relationship between adherence to a Mediterranean diet, measured by the Mediterranean Diet Score, and blood pressure (systolic blood pressure ≥ 130 mmHg, diastolic blood pressure ≥ 85 mmHg).	Logistic regression	Age Sex	OR (95% CI) Systolic blood pressure ≥ 130 mmHg Mediterranean Diet Score 4–5: 0.4 (0.1–1.8) Mediterranean Diet Score 6–8: N/A (*p* = 0.03) Diastolic blood pressure ≥85 mmHg Mediterranean Diet Score 4–5: 0.3 (0.1–1.8) Mediterranean Diet Score 6–8: N/A (*p* = 0.028)	N/A
Smith et al., 2014 [14]	WCRF/AICR guidelines	Associations between meeting WCRF/AICR guidelines and blood pressure.	Log-binomial regression	Age Race CRT (medical records) Education (questionnaires) Smoking status (questionnaires) Age at diagnosis (medical records)	Of the men with hypertension (53.0%), 78.9% did not follow WCRF/AICR guidelines. 40.6% women with high blood pressure; 78.8% did not follow WCRF/AICR guidelines.	N/A
Bérard et al., 2020 [26]	MEDAS KIDMED HDI-2018 HEI-2015 E-DIITM FRAP % UPF	Association between adherence to nutritional scores and blood pressure and hypertension. Pre-hypertension and hypertension were defined, respectively, as blood pressure ≥ 130/85 and <140/90 mmHg in adults and ≥90th and <95th percentile for age and height in children and ≥140/90 mmHg or taking medication in adults and ≥95th percentile for age and height or taking medication in children.	Logistic regression	Sex Survivor time (medical records)	Tertile 2 vs. 1, OR (95% CI) MEDAS: 1.714 (0.55–5.38), *p* = 0.36 KIDMED: 0.117 (0.01–1.28), *p* = 0.08 HDI-2018: 0.945 (0.37–2.42), *p* = 0.91 HEI-2015: 0.500 (0.18–1.42), *p* = 0.19 E-DIITM: 3.029 (1.01–9.11), *p* = 0.049 FRAP: 0.723 (0.27–1.96), *p* = 0.52 % UPF: 0.781 (0.24–2.57), *p* = 0.68 Tertile 3 vs. 1, OR (95% CI) MEDAS: 1.021 (0.25-4.12), *p* = 0.98 KIDMED: 0.275 (0.04–1.72), *p* = 0.17 HDI-2018: 0.425 (0.14–1.31), *p* = 0.14 HEI-2015: 0.821 (0.32–2.10), *p* = 0.68 E-DIITM: 1.135 (0.35–3.71), *p* = 0.83 FRAP: 0.518 (0.17–1.55), *p* = 0.24 % UPF: 1.078 (0.36–3.33), *p* = 0.89 Tertile 2 and 3 vs. 1, OR (95% CI) MEDAS: 1.483 (0.52–4.26), *p* = 0.46 KIDMED: 0.193 (0.04–1.00), *p* = 0.050 HDI-2018: 0.589 (0.25–1.37), *p* = 0.22 HEI-2015: 0.696 (0.31–1.57), *p* = 0.38 E-DIITM: 1.928 (0.68–5.44), *p* = 0.21 FRAP: 0.625 (0.26–1.53), *p* = 0.31 % UPF: 0.934 (0.35–2.53), *p* = 0.89	Tertile 2 vs. 1, OR (95% CI) MEDAS: 1.714 (0.55–5.38), *p* = 0.36 KIDMED: 0.117 (0.01–1.28), *p* = 0.08 HDI-2018: 0.945 (0.37–2.42), *p* = 0.91 HEI-2015: 0.500 (0.18–1.42), *p* = 0.19 E-DIITM: 3.029 (1.00–9.11), *p* = 0.049 FRAP: 0.723 (0.27–1.96), *p* = 0.52 % UPF: 0.781 (0.24–2.57), *p* = 0.68 Tertile 3 vs. 1, OR (95% CI) MEDAS: 1.021 (0.25–4.12), *p* = 0.98 KIDMED: 0.275 (0.04–1.72), *p* = 0.17 HDI-2018: 0.425 (0.14–1.31), *p* = 0.14 HEI-2015: 0.821 (0.32–2.10), *p* = 0.68 E-DIITM: 1.135 (0.35–3.71), *p* = 0.83 FRAP: 0.518 (0.17–1.55), *p* = 0.24 % UPF: 1.078 (0.36–3.24), *p* = 0.89 Tertile 2 and 3 vs. 1, OR (95% CI) MEDAS: 1.483 (0.52-4.26), *p* = 0.46 KIDMED: 0.193 (0.04–1.00), *p* = 0.050 HDI-2018: 0.447 (0.20–1.00), *p* = 0.051 HEI-2015: 0.696 (0.31–1.57), *p* = 0.38 E-DIITM: 1.928 (0.68–5.44), *p* = 0.21 FRAP: 0.609 (0.27–1.39), *p* = 0.24 % UPF: 0.934 (0.35–2.53), *p* = 0.89

Abbreviations: CI, confidence interval; CRT, cranial radiotherapy; E-DIITM, Energy-Adjusted Dietary Inflammatory Index; FRAP, ferric-reducing ability of plasma; HDI, Healthy Diet Indicator; HEI, Healthy Eating Index; IOM, Institute of Medicine; KIDMED, Mediterranean Diet Quality Index for Children and Adolescents; MEDAS, Mediterranean Diet Adherence Screener; N/A, not applicable; OR, odd ratio; % UPF, NOVA classification; WCRF/AICR, World Cancer Research Fund/American Institute for Cancer Research.

**Table 6:** Association between diet and dyslipidaemia indicators.

Study	Exposures/ Interventions	Outcomes	Data Analysis Method	Confounding (Method)	Results			
					HDL-C	LDL-C	Triglycerides	Dyslipidaemia
Landy et al., 2013 [22]	Daily caloric intake Total HEI score	Associations between total daily caloric intake relative to IOM recommendations or HEI scores and LDL and HDL cholesterol.	Multivariate linear regression	Age Sex	Daily caloric intake β = −0.08, *p* = 0.47 Total HEI score β = 0.11, *p* = 0.31	Daily caloric intake β = 0.08, *p* = 0.46 Total HEI score β = −0.02, *p* = 0.81	N/A	N/A
Tonorezos et al., 2013 [25]	Mediterranean Diet Score	The relationship between adherence to a Mediterranean diet, measured by the Mediterranean Diet Score, and HDL-C and triglycerides.	Logistic regression	Age Sex	OR (95% CI) Mediterranean Diet Score 4–5: 0.5 (0.1–1.5) Mediterranean Diet Score 6–8: 0.2 (0.1–0.8) (*p* = 0.01)	N/A	OR (95% CI) Mediterranean Diet Score 4–5: 1.1 (0.4–3.1) Mediterranean Diet Score 6–8: 0.6 (0.2–2.2) (*p* = 0.5)	N/A
Smith et al., 2014 [14]	WCRF/AICR guidelines	Associations between meeting WCRF/AICR guidelines and low HDL and high triglycerides.	Log-binomial regression	Age Race CRT (medical records) Education (questionnaires) Smoking status (questionnaires) Age at diagnosis (medical records)	Among the 42.6% of female with low HDL, 81.6% did not follow the WCRF/AICR guidelines. 38.2% of male with low HDL, 81.7% did not follow the WCRF/AICR guidelines.	N/A	Among the 21% of female with high triglycerides, 76.7% did not follow the WCRF/AICR guidelines. 33.8% of male with high triglycerides, 82.2% did not follow the WCRF/AICR guidelines.	N/A
Morel et al., 2019 [40]	Macronutrient Minerals Vitamins Food groups	Association between macronutrient, minerals, vitamins, food groups intake and low HDL-C in ALL survivors.	Logistic regression	BMI (kg/m^2^) (anthropometric evaluations) Age at diagnosis (years) (medical records) Age at diagnosis squared (years) (medical records) Sex (female) (medical records) Total energy intake (kcal) (FFQ and calculation) Moderate-to-vigorous physical activity (minutes per day) (Minnesota Leisure Time Physical Activity Questionnaire and the Tecumseh Self-Administered Occupational Physical Activity Questionnaire)	Tertile 2 vs. Tertile 1, OR (95% CI) Macronutrients Proteins: 0.300 (0.12–0.74), *p* = 0.009 Carbohydrates: 0.705 (0.29–1.70), *p* = 0.436 Fat: 0.723 (0.30–1.74), *p* = 0.468 Fibre: 0.914 (0.41–2.02), *p* = 0.824 Omega-3: 1.347 (0.59–3.05), *p* = 0.475 Omega-6: 0.897 (0.39–2.10), *p* = 0.800 Ratio omega-3:omega-6: 1.087 (0.48–2.44), *p* = 0.840 Minerals Calcium: 0.774 (0.33–1.80), *p* = 0.553 Magnesium: 0.624 (0.27–1.42), *p* = 0.262 Phosphorus: 0.362 (0.15–0.88), *p* = 0.024 Potassium: 0.754 (0.32–1.79), *p* = 0.523 Sodium: 0.382 (0.15–0.97), *p* = 0.044 Iron: 0.478 (0.21–1.11), *p* = 0.086 Zinc: 0.311 (0.13–0.76), *p* = 0.010 Copper: 0.32 (0.13–0.76), *p* = 0.009 Manganese: 0.616 (0.27–1.39), *p* = 0.243 Selenium: 0.377 (0.16–0.89), *p* = 0.026 Vitamins Retinol: 0.639 (0.28–1.47), *p* = 0.291 Alpha-carotene: 1.444 (0.66–3.16), *p* = 0.356 Beta-carotene: 1.523 (0.67–3.44, *p* = 0.312 Thiamine: 0.634 (0.27–1.51), *p* = 0.302 Riboflavin: 0.300 (0.12–0.74), *p* = 0.009 Niacin: 0.268 (0.11–0.65), *p* = 0.004 Vitamin B6: 0.871 (0.38–2.01), *p* = 0.747 Choline: 0.480 (0.20–1.16), *p* = 0.104 Folic acid: 0.624 (0.26–1.47), *p* = 0.281 Vitamin B12: 0.713 (0.31–1.63), *p* = 0.424 Vitamin C: 0.850 (0.37–1.93), *p* = 0.698 Vitamin D: 0.713 (0.32–1.60), *p* = 0.414 Vitamin K: 1.181 (0.51–2.71), *p* = 0.695 Food groups Meat: 0.572 (0.23–1.40), *p* = 0.222 Fish and seafood: 1.166 (0.49–2.80), *p* = 0.731 Dairy: 0.886 (0.36–2.18), *p* = 0.792 Fat: 1.179 (0.48–2.92), *p* = 0.722 Vegetables: 1.165 (0.44–3.07), *p* = 0.757 Legumes: 1.016 (0.41–2.51), *p* = 0.971 Fruits: 0.261 (0.10–0.70), *p* = 0.008 Fast food: 2.405 (1.03–5.63), *p* = 0.043 Tertile 3 vs. Tertile 1, OR (95% CI) Macronutrients Proteins: 0.289 (0.08–1.00), *p* = 0.05 Carbohydrates: 0.612 (0.17–2.19), *p* = 0.450 Fat: 0.876 (0.26–2.91), *p* = 0.829 Fibre: 0.603 (0.23–1.59), *p* = 0.308 Omega-3: 1.002 (0.40–2.53), *p* = 0.995 Omega-6: 0.652 (0.26–1.61), *p* = 0.354 Ratio omega-3:omega-6: 1.385 (0.62–3.09), *p* = 0.426 Minerals Calcium: 0.830 (0.31–2.22), *p* = 0.711 Magnesium: 0.350 (0.11–1.12), *p* = 0.078 Phosphorus: 0.333 (0.10–1.13), *p* = 0.077 Potassium: 0.692 (0.22–2.18), *p* = 0.52 Sodium: 1.134 (0.35–3.65), *p* = 0.832 Iron: 0.395 (0.12–1.27), *p* = 0.118 Zinc: 0.257 (0.08–0.84), *p* = 0.025 Copper: 0.27 (0.09–0.81), *p* = 0.020 Manganese: 0.639 (0.25–1.60), *p* = 0.340 Selenium: 0.175 (0.05–0.62), *p* = 0.007 Vitamins Retinol: 0.609 (0.24–1.56), *p* = 0.301 Alpha-carotene: 0.880 (0.39–2.00), *p* = 0.760 Beta-carotene: 0.887 (0.37–2.15), *p* = 0.790 Thiamine: 0.741 (0.26–2.11), *p* = 0.575 Riboflavin: 0.248 (0.07–0.86), *p* = 0.028 Niacin: 0.263 (0.08–0.88), *p* = 0.030 Vitamin B6: 0.395 (0.12–1.27), *p* = 0.119 Choline: 0.518 (0.18–1.50), *p* = 0.225 Folic acid: 0.571 (0.20–1.66), *p* = 0.304 Vitamin B12: 0.580 (0.22–1.55), *p* = 0.276 Vitamin C: 0.864 (0.36–2.07), *p* = 0.744 Vitamin D: 0.633 (0.26–1.53), *p* = 0.309 Vitamin K: 0.988 (0.41–2.40), *p* = 0.978 Food groups Meat: 0.277 (0.09–0.83), *p* = 0.022 Fish and seafood: 0.630 (0.24–1.63), *p* = 0.339 Dairy: 1.155 (0.43–3.09), *p* = 0.775 Fat: 1.581 (0.57–4.39), *p* = 0.379 Vegetables: 1.282 (0.46–3.54), *p* = 0.632 Legumes: 0.902 (0.39–2.08), *p* = 0.809 Fruits: 0.920 (0.38–2.24), *p* = 0.854 Fast food: 2.260 (0.85–6.03), *p* = 0.104	N/A	N/A	N/A
Bérard et al., 2020 [26]	MEDAS KIDMED HDI-2018 HEI-2015 E-DIITM FRAP % UPF	Association between adherence to nutritional scores and low HDL-C, high LDL-C, high triglycerides, dyslipidaemia. Dyslipidaemia was defined as having at least one of the following: triglycerides ≥ 1.7 mmol/L (150.6 mg/dL) in adults and ≥1.47 mmol/L (130.2 mg/dL) in children, LDL-C ≥ 3.4 mmol/L (131.5 mg/dL) in adults and ≥ 3.36 mmol/L (129.9 mg/dL) in children, HDL-C < 1.03 in men (39.8 mg/dL), and <1.3 mmol/L (50.3 mg/dL) in women and < 1.03 mmol/L (39.8 mg/dL) in children.	Logistic regression	Sex Survivor time (Medical records)	Tertile 2 vs. 1, OR (95% CI) MEDAS: 0.401 (0.15–1.05), *p* = 0.06 KIDMED: 0.507 (0.09–2.89), *p* = 0.45 HDI-2018: 1.567 (0.73–3.38), *p* = 0.25 HEI-2015: 1.170 (0.56–2.45), *p* = 0.68 E-DIITM: 2.318 (1.04–5.16), *p* = 0.04 FRAP: 0.749 (0.34–1.64), *p* = 0.47 % UPF: 1.410 (0.55–3.64), *p* = 0.48 Tertile 3 vs. 1, OR (95% CI) MEDAS: 0.636 (0.24–1.67), *p* = 0.36 KIDMED: 1.398 (0.31–6.35), *p* = 0.67 HDI-2018: 0.832 (0.37–1.89), *p* = 0.66 HEI-2015: 0.689 (0.31–1.53), *p* = 0.36 E-DIITM: 2.414 (1.04–5.58), *p* = 0.04 FRAP: 0.603 (0.26-1.41), *p* = 0.24 % UPF: 3.885 (1.54–9.80), *p* = 0.004 Tertile 2 and 3 vs. 1, OR (95% CI) MEDAS: 0.500 (0.22–1.14), *p* = 0.10 KIDMED: 0.883 (0.22–3.53), *p* = 0.86 HDI-2018: 1.244 (0.63–2.47), *p* = 0.53 HEI-2015: 0.911 (0.47–1.76), *p* = 0.78 E-DIITM: 2.359 (1.13–4.92), *p* = 0.02 FRAP: 0.682 (0.34–1.39), *p* = 0.29 % UPF: 2.323 (1.02–5.28), *p* = 0.04	No statistically significant association between the seven dietary scores and high LDL-C (no *p* < 0.05). Tertile 2 vs. 1, OR (95% CI) MEDAS: 1.025 (0.41–2.60), *p* = 0.96 KIDMED: 0.379 (0.05–2.93), *p* = 0.35 HDI-2018: 1.087 (0.46–2.60), *p* = 0.85 HEI-2015: 0.729 (0.31–1.72), *p* = 0.47 E-DIITM: 1.200 (0.50–2.89), *p* = 0.68 FRAP: 1.615 (0.62–4.17), *p* = 0.32 % UPF: 0.407 (0.15–1.13), *p* = 0.09 Tertile 3 vs. 1, OR (95% CI) MEDAS: 0.744 (0.26–2.10), *p* = 0.58 KIDMED: 0.571 (0.08–4.02), *p* = 0.57 HDI-2018: 0.726 (0.29–1.81), *p* = 0.49 HEI-2015: 0.705 (0.29–1.69), *p* = 0.43 E-DIITM: 1.183 (0.48–2.93), *p* = 0.72 FRAP: 1.247 (0.47–3.33), *p* = 0.66 % UPF: 0.728 (0.29–1.84), *p* = 0.50 Tertile 2 and 3 vs. 1, OR (95% CI) MEDAS: 1.006 (0.44–2.30), *p* = 0.99 KIDMED: 0.448 (0.08–2.41), *p* = 0.35 HDI-2018: 0.749 0.35–1.61), *p* = 0.46 HEI-2015: 0.675 (0.32–1.41), *p* = 0.30 E-DIITM: 1.192 (0.54–2.62), *p* = 0.66 FRAP: 1.429 (0.60–3.40), *p* = 0.42 % UPF: 0.556 (0.25–1.26), *p* = 0.16	Tertile 2 vs. 1, OR (95% CI) MEDAS: 1.708 (0.55–5.30), *p* = 0.35 KIDMED: 0.628 (0.09–4.28), *p* = 0.64 HDI-2018: 1.198 (0.47–3.03), *p* = 0.70 HEI-2015: 0.705 (0.28–1.79), *p* = 0.46 E-DIITM: 0.937 (0.34–2.59), *p* = 0.90 FRAP: 2.460 (0.86–7.00), *p* = 0.09 % UPF: 2.998 (0.74–12.1), *p* = 0.12 Tertile 3 vs. 1, OR (95% CI) MEDAS: 0.820 (0.22–3.13), *p* = 0.77 KIDMED: 0.701 (0.13–3.89), *p* = 0.68 HDI-2018: 0.607 (0.21–1.73), *p* = 0.35 HEI-2015: 0.459 (0.17–1.23), *p* = 0.14 E-DIITM: 1.658 (0.62–4.41), *p* = 0.31 FRAP: 1.870 (0.59–5.95), *p* = 0.29 % UPF: 5.434 (1.38–21.4), *p* = 0.02 Tertile 2 and 3 vs. 1, OR (95% CI) MEDAS: 1.586 (0.54–4.52), *p* = 0.40 KIDMED: 1.150 (0.23–5.82), *p* = 0.87 HDI-2018: 0.890 (0.39–2.05), *p* = 0.78 HEI-2015: 0.644 (0.28–1.46), *p* = 0.29 E-DIITM: 1.240 (0.52–2.94), *p* = 0.63 FRAP: 2.217 (0.82–5.99), *p* = 0.12 % UPF: 4.021 (1.12–14.5), *p* = 0.03	No statistically significant association between the seven dietary scores and dyslipidaemia (no *p* < 0.05). Tertile 2 vs. 1, OR (95% CI) MEDAS: 0.584 (0.26–1.31), *p* = 0.19 KIDMED: 0.652 (0.18–2.37), *p* = 0.52 HDI-2018: 1.356 (0.69–2.65), *p* = 0.37 HEI-2015: 0.973 (0.50-1.88), *p* = 0.93 E-DIITM: 1.445 (0.75–2.80), *p* = 0.28 FRAP: 1.406 (0.71–2.78), *p* = 0.33 % UPF: 1.089 (0.52–2.30), *p* = 0.82 Tertile 3 vs. 1, OR (95% CI) MEDAS: 0.603 (0.25–1.44), *p* = 0.26 KIDMED: 1.076 (0.33–3.55), *p* = 0.90 HDI-2018: 0.804 (0.41–1.59), *p* = 0.53 HEI-2015: 0.728 (0.37–1.42), *p* = 0.35 E-DIITM: 1.572 (0.79–3.13), *p* = 0.20 FRAP: 1.406 (0.68–2.90), *p* = 0.36 % UPF: 1.983 (0.93–4.21), *p* = 0.08 Tertile 2 and 3 vs. 1, OR (95% CI) MEDAS: 0.653 (0.32–1.33), *p* = 0.24 KIDMED: 1.107 (0.37–3.31), *p* = 0.86 HDI-2018: 1.077 (0.62–1.89), *p* = 0.80 HEI-2015: 0.817 (0.46–1.44), *p* = 0.49 E-DIITM: 1.502 (0.83–2.71), *p* = 0.18 FRAP: 1.406 (0.76–2.61), *p* = 0.28 % UPF: 1.456 (0.76–2.79), *p* = 0.26

Abbreviations: ALL, acute lymphoblastic leukaemia; CI, confidence interval; CRT, cranial radiotherapy; E-DIITM, Energy-Adjusted Dietary Inflammatory Index; FFQ, Food frequency questionnaire; FRAP, ferric-reducing ability of plasma; HDI, Healthy Diet Indicator; HDL-C, high-density-lipoprotein cholesterol; HEI, Healthy Eating Index; IOM, Institute of Medicine; KIDMED, Mediterranean Diet Quality Index for Children and Adolescents; LDL-C, low-density-lipoprotein cholesterol; MEDAS, Mediterranean Diet Adherence Screener; N/A, not applicable; OR, odd ratio; % UPF, NOVA classification; WCRF/AICR, World Cancer Research Fund/American Institute for Cancer Research.

**Table 7:** Association between diet and presence of 2 or more CVD risk factors.

Study	Exposures/ Interventions	Outcomes	Data Analysis Method	Confounding (Method)	Results	
					Presence of 2 or CVD Risk Factors	Metabolic Syndrome
Tonorezos et al., 2013 [25]	Mediterranean Diet Score	The relationship between adherence to a Mediterranean diet, measured by the Mediterranean Diet Score, and metabolic syndrome.	Logistic regression	Age Sex	N/A	OR (95% CI) Mediterranean Diet Score 4–5: 0.9 (0.3–2.7) Mediterranean Diet Score 6–8: 0.1 (0.01–0.9) (*p* = 0.04) For each point higher on the Mediterranean Diet Score, the odds of having the metabolic syndrome fell by 31% (OR 0.69, for each point higher on the Mediterranean Diet Score, adjusted for age and sex (95% CI 0.50, 0.94; *p* = 0.019)).
Smith et al., 2014 [14]	WCRF/AICR guidelines	Association Between WCRF/AICR guidelines <4 and Metabolic Syndrome.	Log-binomial regression models	Age Age at diagnosis (medical records) CRT (medical records) Education (questionnaires) Household income (questionnaires)	N/A	Relative risks (95% CI) Female: 2.4 (1.7–3.3) Male: 2.2 (1.6–3.0)
Belle et al., 2020 [41]	Na intake estimated from FFQ Na intake estimated from spot urine K intake estimated from FFQ K intake estimated from spot urine	Mean sodium (Na) and potassium (K) intake (g/day) in childhood cancer survivors by personal history of CVD and risk factors: (1) “CVD” including heart attack, cardiomyopathy, angina pectoris, atrial fibrillation, arteriosclerosis, stroke, transient ischemic attack (TIA), and/or deep venous thrombosis; (2) “CVD risk factors” including hypertension (repeated high blood pressure measurements or antihypertensive medication treatment), obesity, diabetes mellitus treated with either tablets or insulin, current smoking, and/or high cholesterol defined as treatment with lipid-lowering medications, or (3) “CVD risk-free” if survivors did not report any of these conditions.	ANCOVA	Sex Age at survey ICCC-3 cancer diagnosis (medical records) Education level (questionnaires) Smoking habits (questionnaires) Physical activity (questionnaires) Diet quality (modified AHEI) Alcohol consumption (FFQ)	Mean Na intake based on FFQ (*p* = 0.538) CVD: 2.9 (95% CI 2.8–2.9) CVD risk factors: 2.8 (95% CI 2.8–2.9) CVD risk-free: 2.8 (95% CI 2.8–2.9) Mean K intake based on FFQ (*p* = 0.058) CVD: 2.7 (95% CI 2.5–2.9) CVD risk factors: 2.6 (95% CI 2.4–2.7) CVD risk-free: 2.8 (95% CI 2.7–2.9) Mean Na intake based on morning-fasting spot urine (*p* = 0.017) CVD: 2.7 (95% CI 2.3–3.0) CVD risk factors: 3.3 (95% CI 3.0–3.6) CVD risk-free: 2.9 (95% CI 2.7–3.0) Mean K intake based on morning-fasting spot urine (*p* = 0.490) CVD: 1.3 (95% CI 0.8–1.8) CVD risk factors: 1.7 (95% CI 1.3–2.1) CVD risk-free: 1.6 (95% CI 1.4–1.8)	N/A
Bérard et al., 2020 [26]	MEDAS KIDMED HDI-2018 HEI-2015 E-DIITM FRAP % UPF	Association between adherence to nutritional scores and having ≥2 cardiometabolic complications, including dyslipidaemia, pre-hypertension or hypertension, obesity, and insulin resistance.	Logistic regression	Sex Survivor time (medical records)	Tertile 2 vs. 1, OR (95% CI) MEDAS: 0.800 (0.33–1.97), *p* = 0.63 KIDMED: 0.424 (0.12–1.57), *p* = 0.20 HDI-2018: 1.079 (0.52–2.23), *p* = 0.84 HEI-2015: 1.053 (0.52–2.12), *p* = 0.88 E-DIITM: 2.506 (1.22–5.15), *p* = 0.01 FRAP: 1.509 (0.73–3.13), *p* = 0.27 % UPF: 0.647 (0.29–1.47), *p* = 0.30 Tertile 3 vs. 1, OR (95% CI) MEDAS: 1.380 (0.54–3.50), *p* = 0.30 KIDMED: 0.735 (0.23–2.40), *p* = 0.61 HDI-2018: 1.191 (0.58–2.43), *p* = 0.63 HEI-2015: 0.750 (0.36–1.55), *p* = 0.44 E-DIITM: 1.613 (0.74–3.50), *p* = 0.23 FRAP: 1.245 (0.57–2.73), *p* = 0.58 % UPF: 1.128 (0.51–2.49), *p* = 0.77 Tertile 2 and 3 vs. 1, OR (95% CI) MEDAS: 1.279 (0.58–2.80), *p* = 0.54 KIDMED: 0.728 (0.25–2.13), *p* = 0.56 HDI-2018: 0.728 (0.25–2.13), *p* = 0.56 HEI-2015: 0.911 (0.49–1.68), *p* = 0.77 E-DIITM: 2.076 (1.07–4.07), *p* = 0.03 FRAP: 1.391 (0.71–2.71), *p* = 0.33 % UPF: 0.856 (0.43–1.70), *p* = 0.66	N/A

Abbreviations: AHEI, Alternative Healthy Eating Index; ANCOVA, Analysis of Covariance; CI, confidence interval; CRT, cranial radiotherapy; CVD, cardiovascular disease; E-DIITM, Energy-Adjusted Dietary Inflammatory Index; FFQ, food frequency questionnaire; FRAP, ferric-reducing ability of plasma; HDI, Healthy Diet Indicator; HEI, Healthy Eating Index; ICCC-3, International Classification of Childhood Cancer, 3rd edition; KIDMED, Mediterranean Diet Quality Index for Children and Adolescents; MEDAS, Mediterranean Diet Adherence Screener; N/A, not applicable; OR, odd ratio; % UPF, NOVA classification; WCRF/AICR, World Cancer Research Fund/American Institute for Cancer Research.

**Table 8:** Effects of diet intervention on cardiovascular health in childhood cancer survivors.

Study	Exposures/ Interventions	Data Analysis Method	Outcomes	Results				Confounding (Method)
				Baseline Mean (SD)	Post Intervention Mean (SD)	Difference Mean (95% CI)	*p*-Value	
Zhang et al., 2019 [43]	12-week HEAL intervention: (1) Positive parenting style and practices, (2) Healthy eating, (3) Physical activity	T-test and Chi-square test	BMI *Z*-score	0.79 (1.14)	0.80 (1.26)	0.02 (−0.38–0.41)	0.93	N/A
			BMI percentile	70.3 (28.8)	71.6 (31.7)	1.31 (−10.6–13.3)	0.81	
			Waist circumference	59.5 (6.34)	60.4 (8.0)	0.86 (−1.96–3.68)	0.52	

Abbreviations: BMI, body mass index; CI, confidence intervals; HEAL, Healthy Eating and Active Living; N/A, not applicable; SD, standard deviation.

## Supplementary Materials

The following supporting information can be downloaded at: https://www.mdpi.com/article/10.3390/nu16091315/s1, File S1: PRISMA 2020 Checklist. File S2: Checklist for Analytical Cross Sectional Studies. File S3: Checklist for Quasi-Experimental Studies (non-randomized experimental studies). File S4: Certainty of evidence based on Grading of Recommendations, Assessment, Development and Evaluation (GRADE).
