# Supplementary material for: The Role of Diet in the Cardiovascular Health of Childhood Cancer Survivors—A Systematic Review
Source: Nutrients. 2024 Apr 27;16(9):1315. doi: 10.3390/nu16091315 (PMC11085214; doi:10.3390/nu16091315)
Supplement: Supplementary file 1 [file nutrients-16-01315-s001.zip › S4 File - GRADE.pdf]

S4 File. Certainty of evidence based on Grading of Recommendations, Assessment, Development and Evaluation (GRADE).

	Outcome	Risk of Bias	Imprecision	Inconsistency	Indirectness	Publication Bias	Overall
**Obesity indicators**	BMI	-1	0	-1	0	0	⊕⊕
	Waist circumference	-1	0	-1	0	0	⊕⊕
	Percent body fat	-1	-1	0	0	-1	⊕
	Visceral adiposity and subcutaneous adiposity	-1	-1	0	0	-1	⊕
	Obesity [a]	-1	-1	0	0	0	⊕⊕
**Diabetes indicators**	HOMA-IR	-1	0	-1	0	0	⊕⊕
	Glucose	-1	0	-1	0	0	⊕⊕
	Insulin	-1	-1	0	0	-1	⊕
	Insulin resistance [b]	-1	-1	0	0	0	⊕⊕
**Hypertension indicators**	Blood pressure	-1	0	-1	0	0	⊕⊕
	Hypertension [c]	-1	-1	0	0	0	⊕⊕
**Dyslipidaemia indicators**	HDL-C	-1	0	-1	0	0	⊕⊕
	LDL-C	-1	-1	0	0	0	⊕
	Triglycerides	-1	0	-1	0	0	⊕⊕
	Dyslipidaemia [d]	-1	-1	0	0	0	⊕⊕
**2 or more CVD risk factors**	Presence of 2 or more CVD risk factors	-1	0	0	0	0	⊕⊕⊕

Metabolic syndrome [e]	-1	0	0	0	0	⊕⊕⊕

Abbreviations: BMI: body mass index; HDL-C: high-density lipoprotein-cholesterol; HOMA-IR: homeostatic model assessment – insulin resistance; LDL-C: low-density lipoprotein-cholesterol.

Certainty of Evidence classified as either “very low”, “low”, “moderate”, or “high”. The certainty could be downgraded due to a high risk of bias, inconsistency (unexplained heterogeneity), indirectness (lack of generalisability or external validity), imprecision (small sample size or wide confidence intervals), or the presence of publication bias.

⊕ = very low, ⊕⊕ = low, ⊕⊕⊕ = moderate, ⊕⊕⊕⊕ = high.

[a] Obesity was defined as having at least one of: BMI ≥ 30kg/m^2 in adults and ≥ 97th percentile in children, waist circumference ≥ 102cm in men, ≥ 88cm in women and ≥ 95th percentile in children.

[b] Insulin resistance was defined as having at least one of: blood fasting glucose ≥6.1 mmol/L (109.8 mg/dL), glycated haemoglobin ≥6% and <6.5% and homeostasis model assessment-insulin resistance ≥2.86 in adults and ≥95th percentile in children.

[c] Hypertension were defined, respectively, as: blood pressure ≥130/85 and <140/90 mmHg in adults and ≥90th and <95th percentile for age and height in children and ≥140/90 mmHg or taking medication in adults and ≥95th percentile for age and height or taking medication in children.

[d] Dyslipidaemia was defined as having at least one of: triglycerides ≥ 1.7 mmol/L (150.6 mg/dL) in adults and ≥ 1.47 mmol/L (130.2 mg/dL) in children, LDL-C ≥ 3.4 mmol/L (131.5 mg/dL) in adults and ≥ 3.36mmol/L (129.9 mg/dL) in children, HDL-C < 1.03 in men (39.8 mg/dL), and < 1.3 mmol/L (50.3 mg/dL) in women and < 1.03mmol/L (39.8 mg/dL) in children.

[e] Metabolic syndrome was defined as three or more of the following: 1) abdominal obesity (waist circumference of > 102 cm in males or > 88 cm in females); 2) triglycerides ≥ 150 mg/dL; 3) high-density lipoprotein (HDL) cholesterol < 40 mg/dL in males or < 50 mg/dL in females; 4) hypertension (systolic pressure ≥ 130 mm Hg or diastolic pressure ≥ 85 mm Hg); and 5) fasting plasma glucose ≥ 100 mg/dL, consistently across the studies.
